# Comparative genomic analysis of the MAPKKK gene family reveals WGD-driven expansion and strong evolutionary constraints in Asteraceae

**DOI:** 10.3389/fpls.2026.1791339

**Published:** 2026-03-05

**Authors:** Yang Chen, Zhibin Han, Rongjie Peng, Chun Zhang

**Affiliations:** Department of Agronomy, Hetao College, Bayannur, China

**Keywords:** Asteraceae, conserved gene, *MAPKKK*s, purifying selection, stress-responsive, WGD

## Abstract

Mitogen-activated protein kinase kinase kinases (MAPKKKs) are central components of the MAPK cascade and play crucial roles in plant growth, development, and stress responses. Nevertheless, the evolutionary dynamics and functional diversification of this gene family within the Asteraceae lineage remain poorly characterized. In this study, we conducted a comprehensive comparative genomic analysis of the MAPKKK family across ten representative Asteraceae species. A total of 1,009 *MAPKKK* genes were identified and phylogenetically classified into three subfamilies—RAF, MEKK, and ZIK—which were further subdivided into 14 distinct clusters. Structural analysis revealed considerable variation in intron length and gene architecture among subfamilies and species. All Asteraceae *MAPKKK* genes were grouped into 64 orthologous gene groups (OGGs), including 16 conserved, 28 variable, and 30 rare OGGs. Conserved OGGs comprised 296 genes, accounting for 29.34% of the total *MAPKKK* genes, and were subject to significantly stronger purifying selection compared with dispensable genes. Chromosomal localization and synteny analyses indicated that whole-genome duplication (WGD) events were the primary drivers of MAPKKK family expansion, with conserved genes retained preferentially under stronger selective constraints than those arising from small-scale duplication (SSD) events. Collinearity analysis further showed that conserved genes constituted over 47.91% of syntenic gene pairs, underscoring their high evolutionary conservation. Promoter analysis identified stress-responsive cis-elements as the most abundant category, representing 41.6% of all detected elements, highlighting the potential role of MAPKKKs in environmental adaptation. Transcriptomic and qRT-PCR assays in sunflower under drought, salt, and alkaline stresses revealed several *MAPKKK* genes with distinct and stress-specific expression profiles, such as the conserved genes *Hann_MAPKKK16* and *Hann_MAPKKK135*. Together, these results provide important insights into the evolutionary mechanisms governing MAPKKK family expansion and functional specialization, advancing our understanding of stress adaptation in Asteraceae species.

## 1 Introduction

In sessile organisms like plants, the ability to rapidly perceive and respond to fluctuating environmental conditions is crucial for survival. The mitogen-activated protein kinase kinase kinases (MAPKKKs) serve as highly conserved, primary signal transduction module across eukaryotes, translating extracellular stimuli into intracellular physiological responses (Liang et al., 2022). A typical MAPK cascade consists of three functionally interlinked kinases: MAPK kinase kinases (MAPKKKs), MAPK kinases (MAPKKs), and MAPKs (Plotnikov et al., 2011; Song et al., 2025). Within this hierarchy, MAPKKKs occupy the most upstream position and function as the central command nodes. They govern the specificity and magnitude of signaling fluxes by phosphorylating MAPKKs, which in turn activate MAPKs, ultimately triggering the expression of downstream genes involved in growth, development, and stress adaptation (Zhang and Zhang, 2022).

Structural and phylogenetic analyses in model species, such as *Arabidopsis thaliana* and *Oryza sativa*, have classified the plant MAPKKK superfamily into three distinct subfamilies based on their kinase domain signatures: rapidly accelerated fibrosarcoma (RAF), mitogen extracellular signal-regulated kinase kinase (MEKK), and zipper-interacting kinase (ZIK) (Yongqing Feng et al., 2025). Compared to downstream components, the MAPKKK family is notably larger and more diverse. For instance, there are 80 *MAPKKK* genes in *Arabidopsis thaliana* (Group, 2002), 75 *MAPKKK* genes in rice (Rao et al., 2010), 67 in grape (Y. Feng et al., 2025), 107 in sugarcane (Ali et al., 2025), and 123 in apple (Sun et al., 2017). This extensive expansion is not merely redundant, but provides the raw genetic material for functional divergence and neofunctionalization (Li M. et al., 2022; Shi et al., 2022). In rice *OsMAPKKK28* expression affects drought tolerance by altering drought-related genes and physiological traits like stomatal aperture (Ren et al., 2024). Under salinity and cold stress, MAPKKKs activate MAPK cascades to regulate ionic and osmotic balance, enhancing plant adaptability (Huang et al., 2025; Li W. et al., 2022; Liao et al., 2021; Xing et al., 2024; Yuan et al., 2022). When pathogens attack, MAPKKKs trigger defense responses via the MAPK signaling pathway, boosting disease resistance (He et al., 2021; Li et al., 2021; Xu et al., 2020). MAPKKK could positively contributes to drought and osmotic stress via ABA mediated pathway (Li et al., 2017). MAPKKKs modulate the expression of senescence-associated genes, thereby influencing the initiation and progression of leaf senescence (Xie et al., 2020). Specific expression patterns of *MAPKKK* genes play crucial roles in organ development, including roots, stems, and leaves (Chen et al., 2020; Xi et al., 2021; Zhang et al., 2021). Thus, the expansion of the MAPKKK repertoire is intimately linked to a plant’s adaptive plasticity.

The Asteraceae family, representing the largest and most diverse clade of eudicots, exemplifies exceptional evolutionary success, thriving in habitats ranging from arid deserts to saline-alkaline soils (Huang et al., 2016). Recent genomic insights suggest that this adaptability is largely driven by ancient and recent whole-genome duplication (WGD) (Liu et al., 2020; Sun et al., 2017; Wang et al., 2015). WGDs create massive genetic redundancy, allowing duplicated genes to escape selective pressure and evolve novel functions—a process crucial for stress adaptation (Jiao et al., 2011; Yang et al., 2023). While MAPKKK families have been characterized in various crops, the evolutionary trajectory of this family within Asteraceae lineage remains poorly understood. Specifically, how ancient polyploidy events have shaped the architecture, synteny, and stress-responsive expression patterns of MAPKKKs in this massive family is a question that has not been systematically addressed.

In this study, we performed a comprehensive comparative genomic analysis of the *MAPKKK* gene family across ten representative Asteraceae species, including *Helianthus annuus*, *Lactuca sativa*, *Taraxacum kok-saghyz*, *Arctium lappa*, *Tagetes erecta*, *Cichorium intybus*, *Mikania micrantha*, *Erigeron canadensis*, *Centaurea solstitialis*, and *Rutidosis leptorrhynchoides*. We systematically identified family members and investigated their phylogenetic relationships, gene structure, chromosomal distribution, and syntenic relationships to trace their evolutionary history. Furthermore, focusing on *Helianthus annuus* (sunflower) as a model for stress tolerance, we validated the expression patterns of key *MAPKKK* genes under drought, salinity, and alkaline stresses using qRT-PCR. Our findings provide new insights into how gene family expansion driven by WGD contributes to the environmental adaptability of Asteraceae species.

## 2 Materials and methods

### 2.1 Identification of *MAPKKK* genes

Genome assembly and annotations for ten Asteraceae species were retrieved from NCBI GenBank (**Supplementary Table 1**). The ten Asteraceae species were selected to represent the family’s broad phylogenetic and ecological diversity, encompassing three major subfamilies (Asteroideae, Cichorioideae, and Carduoideae) and diverse life forms, including major crops (*H. annuus*), medicinal plants (*A. lappa*), and invasive weeds (*M. micrantha*). The genome data of *A. thaliana* was obtained from Phytozome v13 (https://phytozome-next.jgi.doe.gov/). To accurately identify MAPKKK family members, a two-step identification strategy was employed.

First, 80 known MAPKKK proteins sequences from *A. thaliana* (MAPK Group, 2002) were used as query sequences to construct a specific Hidden Markov Model (HMM) profile using the hmmbuild tool in the HMMER package (v3.3.2) (Eddy, 2011). Second, the protein sets of 10 Asteraceae species were scanned using kinase domain model (PF00069, E-value cutoff: 1×10^-10^) from Pfam (El-Gebali et al., 2019) via hmmsearch (v3.3.2) (Eddy, 2011).

To ensure the reliability of the candidate genes, all putative MAPKKK gene were identified and validated by InterProScan (Jones et al., 2014) (v5.74-105.0). Sequences lacking a complete kinase domain or those shorter than 100 amino acids were discarded. Additionally, to avoid redundancy, only the longest transcript for each locus was retained for downstream analysis. Finally, the confirmed *MAPKKK* genes were renamed based on their chromosomal positions.

### 2.2 Gene structure characterization

The exon-intron architectural information was retrieved from the genome annotation files of the ten Asteraceae species. Key structural metrics, including total gene length, intron lengths, and intron/CDS numbers, were extracted and computed using custom Perl/Python scripts. These structural characteristics were subsequently used for comparative statistical analysis across different subfamilies.

### 2.3 Phylogenetic reconstruction and subfamily classification

Full-length MAPKKK protein sequences from the ten Asteraceae species and *A. thaliana* were aligned using MAFFT (Nakamura et al., 2018) (v7.490) with the ‘--auto’ parameter. The resulting alignments were trimmed using trimAl (Capella-Gutiérrez et al., 2009) (v1.4.rev22) with a gap threshold of 0.2 (-gt 0.2) to eliminate poorly aligned regions. A maximum likelihood phylogenies tree was constructed by IQ-TREE2 (v2.0.7) (Minh et al., 2020). The best-fit amino acid substitution model was automatically selected by ModelFinder implemented in IQ-TREE2. Branch support values were calculated using 1,000 replicates of the Ultrafast Bootstrap (UFBoot) approximation. For classification, the Asteraceae *MAPKKK* genes were categorized into three subfamilies (RAF, MEKK, and ZIK) based on their phylogenetic clustering with reported *A. thaliana* MAPKKK members.

### 2.4 Systematic comparative genomic analysis of *MAPKKK* family in ten Asteraceae species

To describe the conservation and variation patterns of MAPKKK genes across Asteraceae species, we classified the orthologous gene groups (OGGs) into three categories based on their presence in the ten species (Tong et al., 2025): (i) conserved OGGs (present in all ten Asteraceae species), (ii) variable OGGs (present in nine of species, allowing for single gene loss or assembly gaps), (iii) rare OGGs (present in 1–8 of species, representing lineage-specific variations).

### 2.5 Calculation of selection pressure

To investigate the evolutionary constraints acting on the *MAPKKK* gene family, pairwise alignments were performed for orthologous and paralogous gene pairs. The protein sequences were aligned and back-translated into codon-based nucleotide alignments using ParaAT (v2.0) with default parameters (Zhang et al., 2012). Subsequently, the non-synonymous (*K*a), synonymous (*K*s) substitution rates, and the *K*a/*K*s ratios were computed using KaKs_Calculator (v3.0) (Zhang, 2022), employing the modified Yang-Nielsen model. For OGGs containing multiple gene pairs, the selection pressure was represented by the arithmetic mean of all pairwise *K*a/*K*s values within the group. The value for each cluster was then calculated as the mean of the Ka/Ks values from all its constituent OGGs.

### 2.6 Analysis of gene duplication and syntenic relationships

To elucidate the expansion mechanisms of the *MAPKKK* gene family, the different modes of gene duplication were identified using DupGen_finder (v1.0.0) (Qiao et al., 2019). *A. thaliana* was employed as the outgroup to facilitate the identification of transposed duplications. All *MAPKKK* genes were classified into five categories: whole-genome duplication (WGD)/segmental duplication, tandem duplication (TD), proximal duplication (PD), transposed duplication (TRD), and dispersed duplication (DSD). Genes unclassified by duplication events were designated singletons.

For synteny analysis, inter-species synteny blocks were identified using MCScanX (Wang et al., 2012a) algorithm with minimum ten genes required to call a collinear block. The inter-species syntenic relationships were investigated and visualized using python package JCVI (v1.5.11) (Tang et al., 2024).

### 2.7 Promoter analysis of the *MAPKKK* gene family

For the identification of promoter regions of the *MAPKKK* gene family in ten Asteraceae species, genomic sequences spanning 2000 bp upstream of the translation initiation site (ATG) of each *MAPKKK* gene were extracted from the corresponding reference genome. The retrieved sequences were then submitted to the PlantCARE database (https://bioinformatics.psb.ugent.be/webtools/plantcare/html/) for *de novo* prediction and annotation of cis-acting regulatory elements. All identified cis-acting elements were further categorized into four classes (stress response, light response, hormone response, and plant growth and development) based on the criteria described in previous studies (Zheng et al., 2025). To evaluate the differences in element content among different subfamilies, the proportion of each individual element relative to the total number of elements within each subfamily was calculated. For comparisons at the orthologous gene group (OGG) level, the proportion of each element was determined relative to the total number of elements in the corresponding OGG. The proportional data of cis-acting elements were visualized as heatmaps using the ggplot2 package (v3.4.4) in R software (v4.3.2).

### 2.8 Transcriptomic expression analysis under drought stress

To investigate the transcriptional responses of *H. annuus MAPKKK* genes to environmental cues, raw RNA-seq datasets related to drought stresses were retrieved from the NCBI Sequence Read Archive (SRA) database source from PRJNA1041959 (Shi et al., 2024). In this project, *H. annuus* seedlings were treated with 15% PEG-6000 to simulate drought stress, and leaf samples were collected for RNA-seq analysis.

The raw reads were subjected to quality control using fastp v0.23.4 (Chen, 2025) and to remove low-quality bases and adapters. The clean reads were mapped to the *H. annuus* reference genome using HISAT2 v2.2.1 (Kim et al., 2015). Gene expression levels were quantified using StringTie v 2.2.3 (Pertea et al., 2015) and normalized as Fragments Per Kilobase of transcript per Million mapped reads (FPKM). For visualization, the row-scale expression heatmaps were generated using Evolview v3 (Subramanian et al., 2019).

### 2.9 Plant cultivation, treatments, RNA isolation, and qRT-PCR

Four-week-old sunflower seedlings were subjected to various abiotic stress treatments. These treatments included drought stress (15% PEG 6000), salt stress (250 mM NaCl), salt-alkali stress (150 mM NaCl + 50 mM Na_2_CO_3_). Leaf samples were collected at 1, 3, 6, 12 and 24 h after treatment initiation and immediately frozen in liquid nitrogen, with samples collected at 0 h (before treatment) served as the control. All samples were stored at − 80 °C until RNA extraction. Total RNA was isolated from leaf tissues using TaKaRa MiniBEST Plant RNA Extraction Kit (Code No.9769), according to the manufacturer protocol. First-strand cDNA was synthesized from 1 µg of total RNA using PrimeScript™ II 1st Strand cDNA Synthesis Kit (Code No.6210A, TaKaRa), RT-qPCR was carried out in TB Green^®^ Premix Ex Taq™ II FAST qPCR (Code No. CN830A, TaKaRa). The *ACTIN* gene was set as an internal reference for normalization, and relative expression was calculated using the 2^−ΔΔCt^ method (Livak and Schmittgen, 2001). All primer sequences used in this study were listed in **Supplementary Table 2**.

## 3 Results

### 3.1 Identification, and phylogenetic analysis of *MAPKKK* genes in Asteraceae

To elucidate the evolutionary history of the *MAPKKK* gene family in Asteraceae, we identified 1,009 *MAPKKK* genes from 10 Asteraceae species (**Supplementary Table 3**) and constructed a phylogenetic tree together with 80 MAPKKKs from *A. thaliana*. Based on the topological structure and established classification in *A. thaliana*, the Asteraceae *MAPKKK* genes were classified into three major subfamilies, including RAF, MEKK, and ZIK (**Supplementary Figure 1**). The *A. thaliana* members within each subfamily clustered distinctly as expected, validating the reliability of our phylogenetic reconstruction. Moreover, MAPKKK orthologs from each Asteraceae genome co-clustered with their *A. thaliana* homologs in nearly all clades, indicating that the diversification of these three subfamilies predates the divergence between Brassicales and Asteraceae.

**Figure 1:**
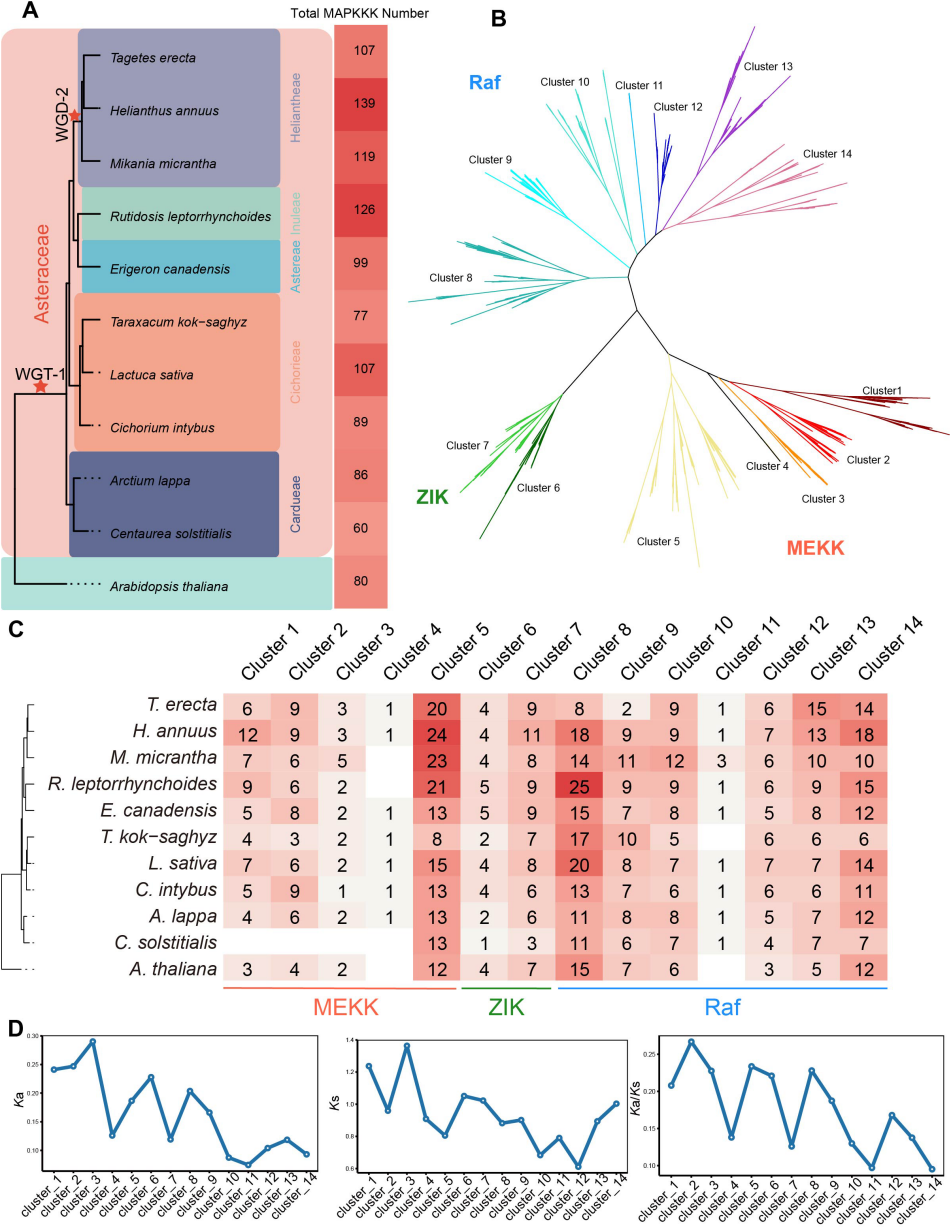
Identification and phylogenetic analysis of *MAPKKK* gene family in ten Asteraceae species. **(A)** The phylogenetic relationships among species are shown on the left, with star symbols indicating whole-genome duplication **(WGD)** or triplication **(WGT)** events. The right panel displays the total number of *MAPKKK* genes per species. **(B)** Maximum Likelihood phylogenetic tree of *MAPKKK* genes from ten Asteraceae species. Subgroups classification of each MAPKKK subfamily based on the tree topology and 14 clusters were obtained. **(C)** Distribution of MAPKKKs across the 14 clusters. **(D)** Comparison of average *K*a, *K*s, and *K*a/*K*s values across different clusters.

The size of the MAPKKK gene family exhibits substantia interspecific variation, ranging from 60 genes in *C. solstitialis* and 139 in *H. annuus* (**Figure 1A**). A contraction was observed in *C. solstitialis*, whereas species within the Heliantheae and Inuleae generally harbor over 100 *MAPKKK* genes. In contrast, species in the Astereae, Cichorieae and Cardueae generally contained fewer than 100 genes, with the exception of *L. sativa* (107 genes). A significant positive correlation was observed between MAPKKK family size and the total number of annotated genes in the genome (Pearson correlation coefficient *r* = 0.69, *p* = 0.018) (**Supplementary Figure 2**). Regarding subfamily composition, the RAF subfamily was the most abundant, comprising 51.40% (*T. erecta*) to 71.67% (*C. solstitialis*) of total *MAPKKK* genes, followed by MEKK (21.67% in *C. solstitialis* to 36.45% in *T. erecta*) and ZIK (6.67% in *C. solstitialis* to 14.15% in *E. canadensis*) (**Supplementary Table 3**).

To gain finer resolution of the evolutionary relationships, the phylogenetic tree was further partitioned into 14 distinct clusters (**Figure 1B**; **Supplementary Table 3**). Clusters 1–5 corresponded to the MEKK subfamily, 6–7 to ZIK, and 8–14 to RAF. Notably, nine of 14 clusters contain members from all ten Asteraceae species, and no cluster is exclusive to a single species (**Figure 1C**). This distribution pattern suggests that the fundamental architecture of the MAPKKK family was established in the common ancestor of the Asteraceae. In addition, we characterized selection pressure profiles of distinct MAPKKK clusters by computing Ka/Ks ratios of orthogroup pairs from the MEKK, ZIK, and RAF subfamilies (**Figure 1D**; **Supplementary Figure 3**). Clusters displayed divergence in selection pressure: MEKK cluster 2 had the highest average Ka/Ks ratio (0.267), while RAF cluster 14 had the lowest (0.095), a nearly threefold difference. This indicates that RAF cluster 14 undergoes stronger purifying selection to maintain highly conserved functions, whereas MEKK cluster 2 experiences weaker selection and may retain greater potential for functional diversification.

### 3.2 Structural characteristics of *MAPKKK* genes in the Asteraceae

**Figure 2:**
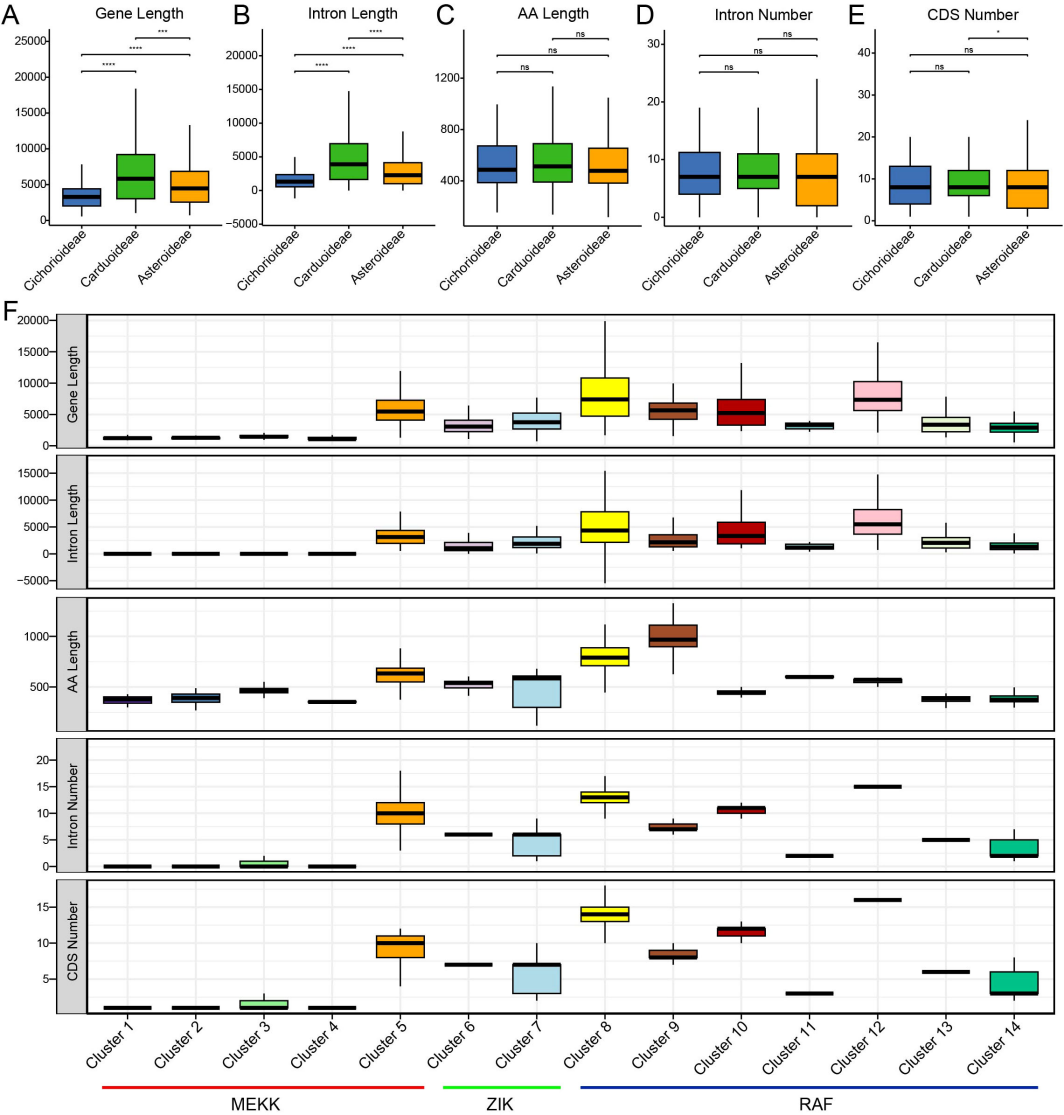
Structural characteristics of *MAPKKK* gene family members in ten Asteraceae species. Boxplot gene length, intron length, Amino acid (AA) length, intron number and CDS number among three clades **(A-E)** and different clusters of each MAPKKK subfamilies **(F)**.

To characterize the structural evolution of the MAPKKK family, we analyzed the exon–intron organization across the ten Asteraceae species (**Figure 2**). At the genomic level, *MAPKKK* genes were found to be significantly longer in Carduoideae compared to the other two lineages Asteroideae and Cichorioideae (**Figure 2A**). This length difference is largely due to an expansion in intron size, rather than an increase in the length of coding regions (**Figure 2B**). No significant differences were observed in coding sequence (CDS) length, intron number, or amino acid sequence length among the three lineages (**Figures 2C–E**), suggesting that the coding capacity of MAPKKKs has remained stable during Asteraceae divergence.

Furthermore, substantial structural divergence was evident among MAPKKK subfamilies and clusters (**Figure 2F**). The RAF subfamily exhibited the most complex and extensive gene structure, with median length of 4,872 bp, significantly exceeding those of the MEKK (3,147 bp) and ZIK (3,373 bp) subfamilies. This expansion in RAF genes, particularly pronounced in Clusters 8 and 12, was driven primarily by increased intron length (median: 4,605 bp and 5,742 bp, respectively) rather than by the elongation of coding regions. However, MEKK subfamily exhibits a distinct intron-poor architecture. Notably, over 80% of genes in MEKK Clusters 1–4 are intronless (single-exon) genes with a median length of only 1,291.5 bp. This structural simplicity contrasts sharply with MEKK Cluster 5 (median 10 introns) and the RAF/ZIK subfamilies (median 6–15 introns).

### 3.3 Conservation and diversity analysis of MAPKKK family in Asteraceae plants

**Figure 3:**
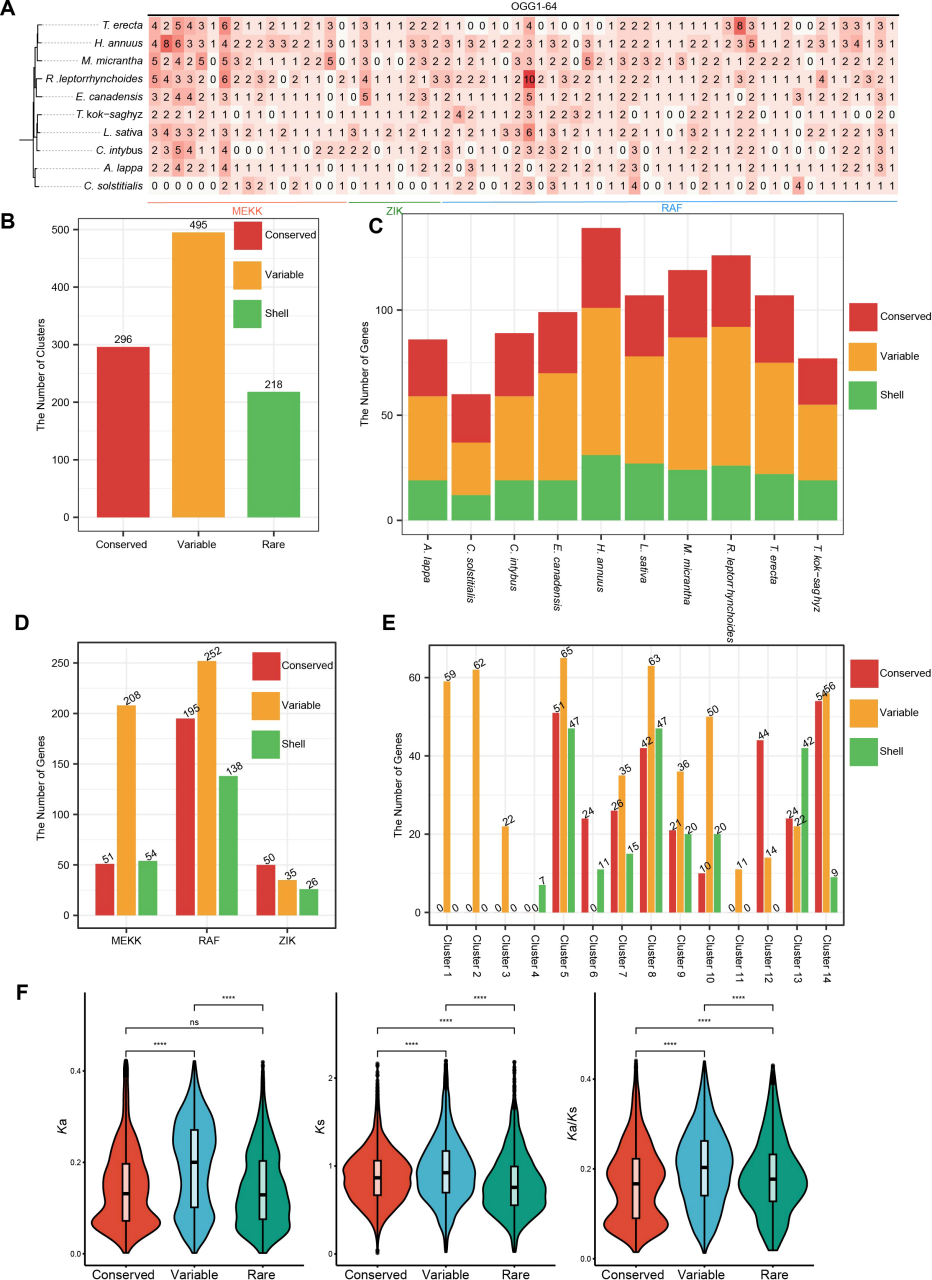
Classification and orthologous variation of the *MAPKKK* gene family in ten Asteraceae species. **(A)** Heatmap illustrating the copy number variation of 64 orthologous gene groups across the ten species. Orthologous gene groups are arranged according to their subfamily classification and cluster identity. The color intensity represents the number of gene copies. **(B)** Statistical distribution of *MAPKKK* genes across three categories. The numbers above the bars indicate the total gene count in each category. **(C)** Composition of conserved, variable, and rare genes within the genome of each examined species. **(D)** Distribution of categories across the three subfamilies. **(E)** Distribution of categories across the 14 clusters. **(F)** Comparison of *K*a, *K*s, and *K*a/*K*s values between conserved, variable, and rare MAPKKKs. Statistical significance was assessed using a Wilcox test. Asterisks indicate statistical significance (*P* < 0.0001).

To explore the evolutionary conservation and diversity of the MAPKKK family across the ten Asteraceae species, we classified the 64 identified orthologous gene groups (OGGs) into categories (**Figure 3A**; **Supplementary Table 3**). The conserved and variable OGGs collectively comprise 16 and 28, respectively, containing 296 (29.34% of total genes) and 495 (49.06% of total genes) genes respectively (**Figure 3B**). This predominance of conserved and variable genes underscores the fundamental conservation of conserved MAPKKK functions across Asteraceae. The distribution of these categories within each species is consistent, with conserved/variable genes constituting the majority of the repertoire (**Figure 3C**). Notably, Clusters 1, 2, 3, and 4 (belonging to MEKK) completely lack conserved genes. This is primarily driven by the extensive gene loss observed in *C. solstitialis*, which lacks orthologs in almost all MEKK clusters (**Figures 3A**, bottom row). Similarly, Cluster 11 lacks conserved genes, which corresponds to the specific absence of this cluster in *T. kok-saghyz* and *C. solstitialis*. These findings indicate that the variable presence of these *MAPKKK* genes across species.

At the subfamily level, distinct conservation patterns were observed (**Figure 3D**). The RAF subfamily contributes the highest number of conserved genes (195 genes) and variable genes (252 genes), reflecting its dominant role and high retention rate during evolution. The ZIK subfamily, although smaller in size, maintains a relatively high proportion of conserved genes (50 conserved vs. 26 rare), suggesting stable inheritance. In contrast, the MEKK subfamily shows a higher degree of variability; it contains 51 conserved genes and 54 rare genes, but a substantial number of variables (208). Further analysis of the 14 clusters revealed the source of this variability (**Figure 3E**).

To assess the natural selection pressures acting on Asteraceae *MAPKKK* genes, we calculated the *K*a, *K*s, and *K*a/*K*s ratios for each OGG (**Figure 3F**). When comparing selection pressures between conserved and dispensable (variable and rare) *MAPKKK* genes, dispensable *MAPKKKs* showed significantly higher median *K*a/*K*s ratios values than conserved MAPKKKs, suggesting that dispensable *MAPKKKs* generally experience relaxed selection pressures, whereas conserved *MAPKKKs* appear to be under stronger purifying selection constraints.

### 3.4 Gene duplication of the *MAPKKK* family in Asteraceae plants

**Figure 4:**
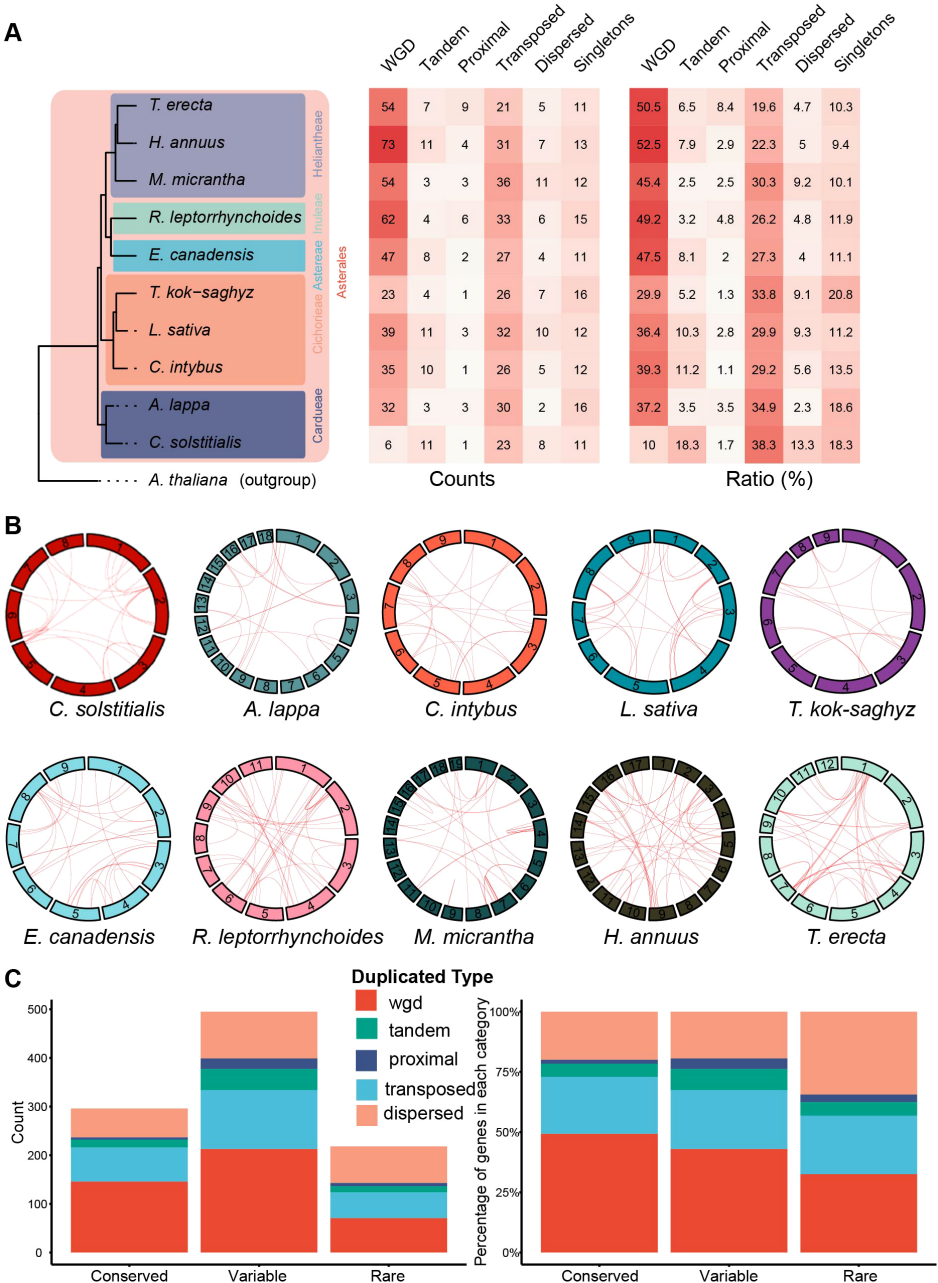
Modes of gene duplication of *MAPKKK* genes in ten Asteraceae plants. **(A)** Heatmap and statistical summary of five gene duplication modes across ten Asteraceae species. The left panel shows the species phylogeny. The middle table displays the absolute number of genes derived from whole-genome duplication (WGD), Tandem (TD), Proximal (PD), Transposed (TRD), and dispersed (DSD) duplications. The right table presents the proportional contribution of each mode. Darker red indicates higher values. **(B)** Circos plots illustrating the chromosomal distribution and syntenic relationships of MAPKKK genes in the ten studied species. The outer circle represents chromosomes (labeled with numbers), and the inner red lines connect paralogous gene pairs originating from WGD/segmental duplication events. **(C)** Distribution patterns of distinct duplicated genes among conserved and non-conserved categories. The left figure displays the quantitative distribution, while the right figure presents the proportional distribution.

To elucidate the expansion mechanisms underlying the MAPKKK family, we identified five distinct duplication modes: whole-genome duplication (WGD), tandem duplication (TD), dispersed duplication, proximal duplication, and transposed duplication (TRD). In nine out of the ten studied species, 23 to 73 *MAPKKK* genes, accounting for ≥30% of the family, were primarily attributed to whole-genome duplication (WGD) events, while tandem duplications contributed to less than 12% (**Figure 4A**; **Supplementary Table 4**). Chromosomal distribution patterns provide strong support, showing widespread paralogous pairs across chromosomes that reflect ancient polyploidization (**Figure 4B**). However, a distinct evolutionary trajectory was observed in *C. solstitialis*. This species retains only 6 WGD-derived genes (10%) but exhibits a high proportion of tandem duplication (TD, 18.3%) and transposed duplications (TRD, 38.3%) (**Figure 4A**).

In addition, we investigated the association between gene duplication events and conserved, variable, and rare genes (**Figure 4C**). Statistical analysis revealed differences in the proportion of whole-genome duplication (WGD)-derived genes among different gene categories: conserved genes harbored the highest proportion of WGD-derived genes (49.32%), followed by variable genes (43.03%), while rare genes contained the lowest proportion (32.57%). This distribution pattern suggests that the highly conserved functional and sequence characteristics of conserved genes may be primarily driven by WGD events. In contrast, non-conserved genes (e.g., rare genes) were predominantly contributed by other duplication types, with dispersed duplication-derived genes accounting for 34.40%—a proportion higher than that in conserved genes (19.93%).

### 3.5 Selection pressure links duplication type and conservation in MAPKKK family

To quantify the selective pressures acting on the *MAPKKK* gene family, we calculated Ka/Ks ratios for all intraspecific paralogous pairs across 10 Asterales species (**Supplementary Figure 3**). The analysis revealed pervasive purifying selection across the Asteraceae family, with most species exhibiting a Ka/Ks ratio ≤ 0.4. This indicates that pervasive purifying selection and strong functional constraints act on the *MAPKKK* gene repertoire. Additionally, Ks distribution analysis showed that the peaks for three species (*T. erecta*, *H. annuus* and *M. micrantha*) were lower than those of other species, consistent with the occurrence of more recent whole-genome duplication (WGD) events in these lineages than others.

**Figure 5:**
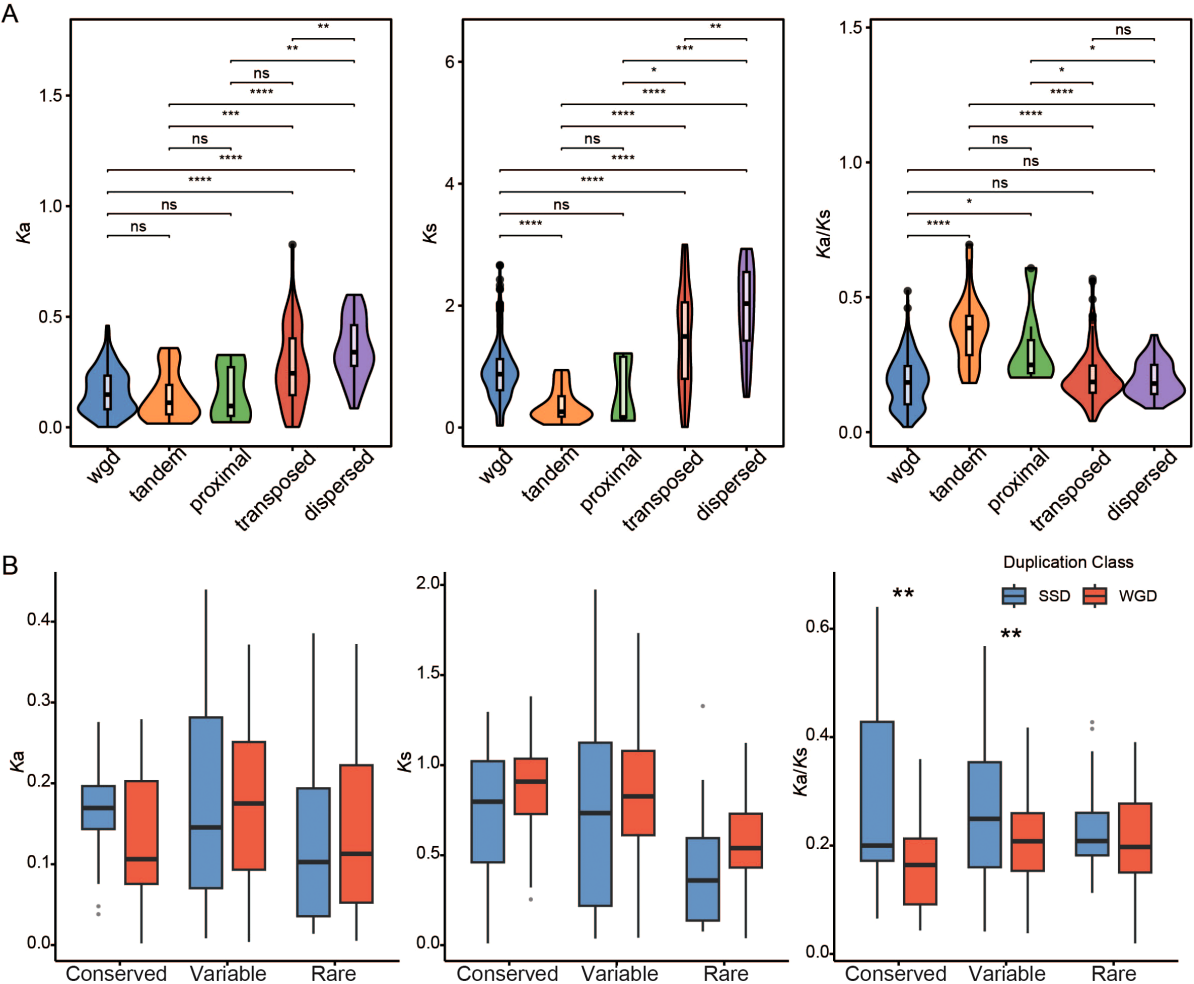
Selection pressure analysis between duplication type and functional conservation in the MAPKKK family. **(A)** Comparison of *K*a, *K*s, and *K*a/*K*s values among different duplication types. **(B)** Comparison of different duplication types for conserved, variable, and rare *MAPKKKs*. Statistical significance was assessed using a Wilcox test. * represents *P* < 0.05, ** represents *P* < 0.01, *** represents *P* < 0.001, **** represents *P* < 0.0001.

To further investigate how different duplication events influence the evolutionary selection pressures on the *MAPKKK* gene family, we performed a combined analysis of Ka/Ks ratios for paralogous pairs categorized by duplication type (**Figure 5A**). Our results revealed significant differences in selection pressures among duplication types: WGD-derived paralogous pairs had the lowest average *K*a/*K*s ratio (0.186), which was significantly lower than that of tandem duplication (TD)-derived pairs (0.379) and proximal duplication-derived pairs (0.311). This suggests that duplicated genes generated by WGD events are subject to stronger purifying selection, maintaining higher functional conservation. When integrated with gene conservation analysis (**Figure 5B**), we found that WGD-derived pairs within conserved and variable genes had significantly lower *K*a/*K*s ratios than those from small-scale duplications (SSD). In contrast, no significant differences in *K*a/*K*s ratios were observed among different duplication types within rare genes. These findings reveal a coevolutionary relationship between duplication type and gene functional conservation. WGD events tend to retain gene copies with core functions, while other duplication types provide evolutionary raw materials for functional diversification within the *MAPKKK* gene family.

### 3.6 Collinearity map of the *MAPKKK* gene family across ten Asteraceae species

**Figure 6:**
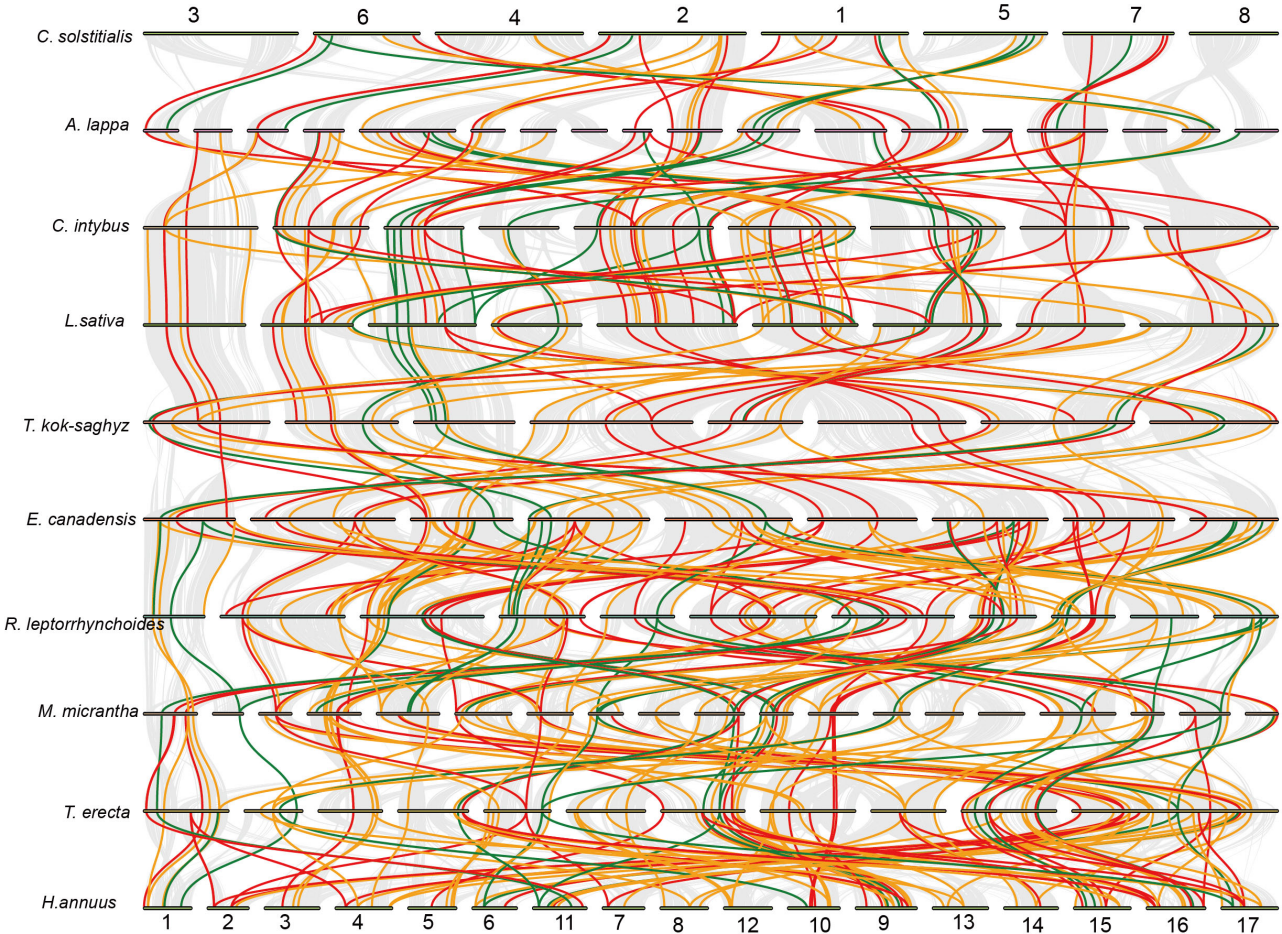
Collinearity analysis of *MAPKKK* gene families in ten Asteraceae plants. Syntenic MAPKKK gene pairs were distinguished by line colors based on gene conservation categories: conserved genes are connected by red lines, variable genes by yellow lines, and rare genes by green lines.

To investigate the evolutionary relationships of the *MAPKKK* gene family in Asteraceae, we performed a pairwise collinearity analysis among the ten Asteraceae genomes (**Figure 6**). Variable collinearity patterns of *MAPKKK* genes among different Asteraceae species, with the number of collinear pairs ranging from 32 (*A. lappa* vs. *C. solstitialis*) to 112 (*H. annuus* vs. *T. erecta*) (**Supplementary Table 5**). Notably, the proportion of conserved genes in these collinear pairs was significantly elevated, reaching 47.91%—a value much higher than the overall proportion of conserved genes (29.34%) in the entire gene family. In contrast, the proportion of rare genes in collinear pairs was reduced from 21.61% (overall proportion) to 18.30% (**Supplementary Table 5** and **Figure 3B**). These findings indicate the core genomic architecture of the *MAPKKK* family remains highly collinear.

### 3.7 Promoter analysis of the *MAPKKK* gene family

**Figure 7:**
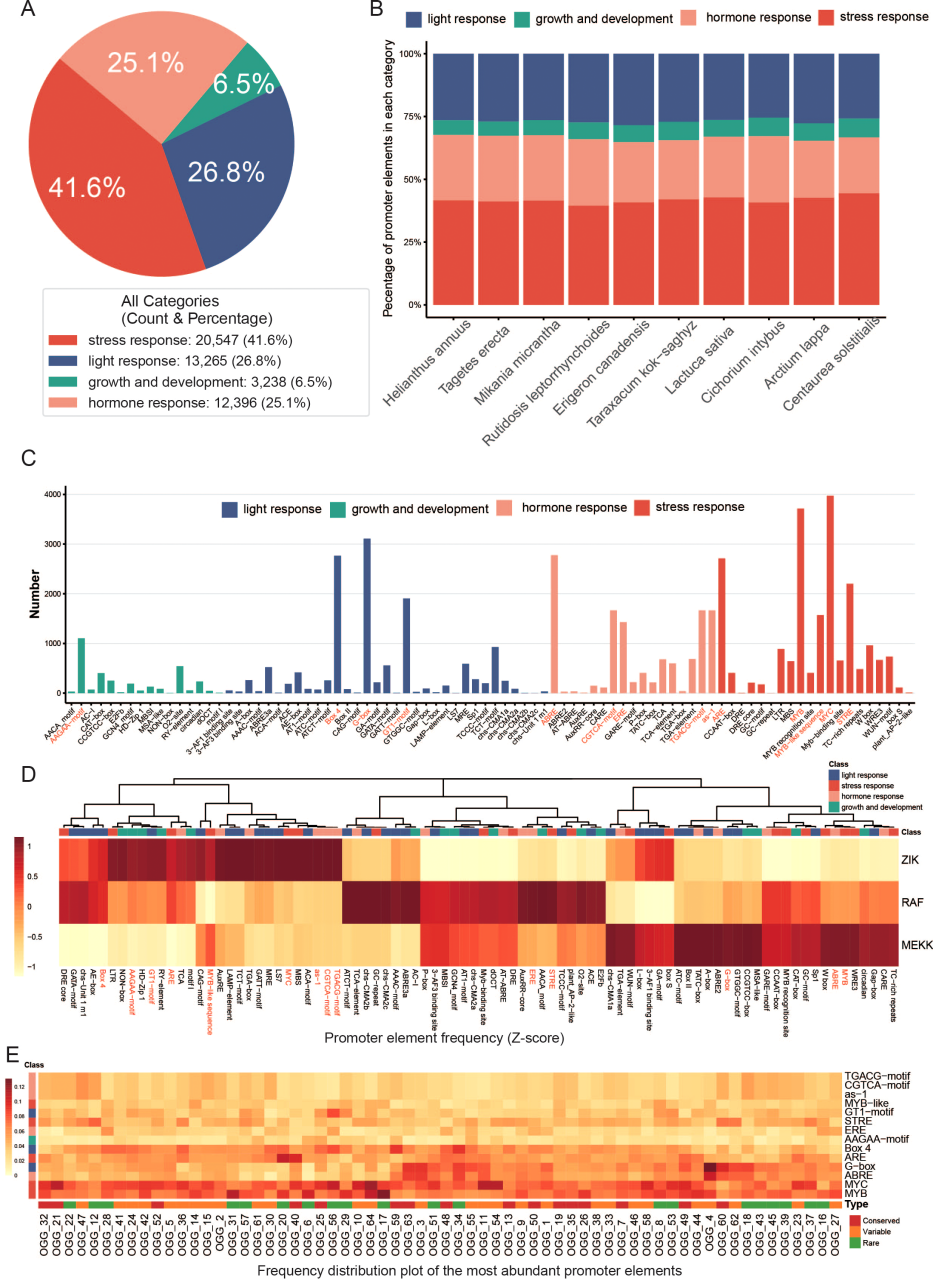
Distribution of the promoter elements of *MAPKKK* genes in each category among ten Asteraceae plants. **(A)** Distribution of all identified promoter elements across functional categories. A supplementary table is included to detail the count and percentage of elements within each category. **(B)** Stacked bar chart illustrating the proportional distribution of promoter elements (stratified by functional category) across distinct Asteraceae species. **(C)** Grouped bar chart depicting the quantitative distribution of individual promoter element types; elements highlighted in red correspond to the most frequently distributed ones in this panel. **(D)** Column-scaled clustered heatmap representing the relative abundance of promoter elements across MAPKKK subfamilies (ZIK, RAF, MEKK). Rows denote MAPKKK subfamilies, columns represent promoter element types, and the color gradient corresponds to the Z-score of element frequency. **(E)** Frequency distribution heatmap illustrating the frequency profile of the most abundant promoter elements for each OGG.

A total of 49,446 cis-acting element instances, belonging to 86 distinct types, were identified from 1,009 *MAPKKK* genes across ten Asteraceae species (**Supplementary Table 6**). Based on their functional annotations, these cis-acting elements were classified into four major groups: stress response (20,547 elements), light response (13,265 elements), hormone response (12,396 elements), and plant growth and development (3,238 elements) (**Figure 7A**). Notably, stress response elements were the most abundant, accounting for 41.6% of the total, indicating that stress adaptation may be a core functional focus of the Asteraceae MAPKKK family. Analysis of element distribution across individual species revealed that all four functional element groups were enriched in the *MAPKKK* promoters of the species, with relatively conserved proportional distributions (**Figure 7B**). This conservation suggests that the basic transcriptional regulatory patterns of *MAPKKK* genes are highly conserved among Asteraceae plants.

Further analysis of high-frequency elements (with over 1,000 occurrences) revealed 14 elements, which were classified into four categories: stress response (MYC, MYB, ARE, STRE, MYB-like), light response (Box−4, G−box, GT1−motif), hormone response (ABRE, ERE, CGTCA−motif, TGACG−motif, as−1), and plant growth and development (AAGAA−motif) (**Figure 7C**). Subfamily-specific analysis uncovered distinct differences in element abundance among MEKK, RAF, and ZIK subfamilies (**Figure 7D**). Specifically, the MEKK subfamily was enriched in MYB, G-box, and ABRE elements; the RAF subfamily contained higher levels of ERE and STRE elements; and the ZIK subfamily was characterized by abundant MYC, CGTCA-motif, TGACG-motif, and as-1 elements. Additionally, Box-4 and ARE elements were highly abundant in both the RAF and ZIK subfamilies.

Finally, we examined the distribution of these high-frequency elements among orthogroups (OGGs). Stress-response elements exhibited distinct, OGG-specific distribution patterns (**Figure 7E**): MYB elements were more abundant in OGG17 and OGG31, both of which are enriched in rare genes; MYC elements were notably enriched in OGG21 (containing conserved genes) as well as in OGG64, OGG1, and OGG54 (each containing variable genes); and ARE elements were predominantly concentrated in OGG40 (conserved genes) and OGG20 (rare genes). Among light-response elements, the G-box was more abundant in OGG4 (variable genes), whereas the GT1-motif was enriched in OGG56 (rare genes). For hormone-response elements, ABRE was highly represented in OGG63 (conserved genes) and OGG4 (variable genes). The plant growth and development-related AAGAA-motif was most abundant in OGG25, which contains conserved genes.

### 3.8 Transcriptomic profiling of *H. annuus MAPKKK* genes under diverse stress response

Given that *MAPKKK* genes function as pivotal intracellular transducers in drought, salinity, and alkaline stress signaling pathways (Nakagami et al., 2005; Xie et al., 2023), and our preliminary analysis identified multiple stress-responsive cis-acting elements in their promoter sequences (**Figure 7A**), we used sunflower (*Helianthus annuus*) as the research material to screen for potential stress-responsive genes based on drought stress leaf transcriptome data(**Supplementary Figure 4**). The results showed that after drought stress treatment, the expression levels of 25 sunflower *MAPKKK* genes changed significantly (|log_2_FC| > 1). These genes were distributed across the three subfamilies: 7 MEKK subfamily genes (including 3 conserved genes and 4 variable genes), 15 RAF subfamily genes (including 4 conserved genes, 5 variable genes, and 6 rare genes), and 3 ZIK subfamily genes (including 1 conserved gene, 1 variable gene, and 1 rare gene) (**Supplementary Table 7**).

**Figure 8:**
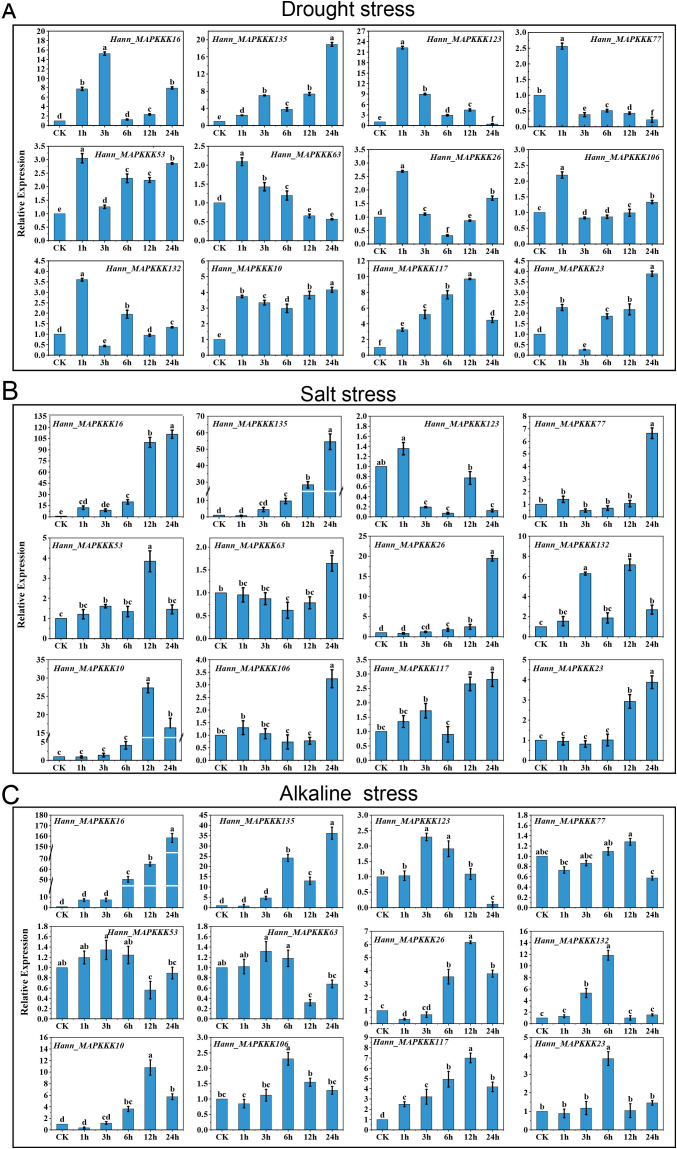
Expression profiles of MAPKKK genes under drought, salt and alkaline stress. Relative expression levels of candidate genes in *H. annuus* leaf treated with drought stress **(A)**, salt stress **(B)** and alkali stress **(C)** for 0, 1, 3, 6, 12, and 24 h. The y-axis represents the relative fold change normalized to the control (CK). Error bars represent the SD of three biological replicates. Different letters indicate significant differences (*P* < 0.05).

To validate the reliability of the transcriptome data and characterize the stress-responsive profiles of key genes under multiple stress conditions, we selected 12 highly expressed genes from this set for quantitative real-time PCR (qRT-PCR) analysis (**Figure 8**). Specifically, we analyzed the expression dynamics of these genes at different time points (0 h, 1 h, 3 h, 6 h, 12 h, and 24 h) under drought stress, and further extended our investigation to their expression patterns under salinity and alkaline stress conditions. Under drought treatment (**Figure 8A**), most genes exhibited rapid response within the first hour. The most prominent early responder was a variable *MEKK* gene, *Hann_MAPKKK123*(*LOC110938159*), which surged to a >21-fold peak at 1 hour, significantly higher than other genes at this time point. Similarly, three RAF genes, *Hann_MAPKKK77* (*LOC110915220*), *Hann_MAPKKK53*(*LOC110890741*), and *Hann_MAPKKK132* (*LOC110940996*), showed immediate upregulation (2.6–3.7 fold) at 1h, suggesting their involvement in early drought signal perception. In contrast, one conserved *ZIK* gene, *Hann_MAPKKK135* (*LOC110941748*), displayed a continuous upward trend, peaking at 24 hours with a remarkable >18-fold increase. The conserved *RAF* gene *Hann_MAPKKK117* (*LOC110935989*) also showed a delayed peak at 12 hours (~10-fold). Conserved *MEKK* gene *Hann_MAPKKK16* (*LOC110877056*) acted as a bridge, peaking at 3 hours (>15-fold) and maintaining high levels, distinguishing it from the transient early responders.

The salt stress response elicited the most widespread high-magnitude induction among the tested genes (**Figure 8B**). Besides the conserved *MEKK* gene *Hann_MAPKKK16* (*LOC110877056*) (>105-fold), the conserved *ZIK* gene, *Hann_MAPKKK135* (*LOC110941748*) again showed exceptional inducibility, peaking at ~54-fold at 24 hours. One rare *ZIK* gene, *Hann_MAPKKK10* (*LOC110869562*) (~27-fold) and *Hann_MAPKKK26* (*LOC110879474*) (>23-fold) also exhibited dramatic upregulation at 12–24 hours. Interestingly, the variable *RAF* gene *Hann_MAPKKK77* (*LOC110915220*), which was an early responder in drought, acted as a late responder in salinity (peaking at 24h), indicating stress-specific regulatory mechanisms.

The response to alkaline stress was particularly robust for specific members of the MAPKKK family (**Figure 8C**). The most striking induction was observed for the conserved MEKK gene *Hann_MAPKKK16* (*LOC110877056*), which exhibited dramatic, continuous upregulation, reaching an exponential peak at 24 hours with a >158-fold increase compared to the control. Similarly, the conserved *ZIK* gene *Hann_MAPKKK135* (*LOC110941748*) showed a >36-fold increase, while the rare *ZIK* gene *Hann_MAPKKK10* (*LOC110869562*) was significantly upregulated (>10-fold) at 12–24 hours, indicating that these genes play critical roles in coping with high pH stress. Additionally, the conserved *RAF* gene *Hann_MAPKKK132* (*gene-LOC110940996*) displayed an >11-fold increase at 6 hours.

Collectively, qRT-PCR results reveal distinct functional specialization in the HaMAPKKK family. Conserved *MEKK* gene *Hann_MAPKKK16* serves as a hypersensitive, multi-stress hub with high expression, while conserved *ZIK* gene *Hann_MAPKKK135* acts as a critical late-stage adaptation gene. Other members show stress-specific roles: *Hann_MAPKKK123* (variable *MEKK*) is drought-specific, *Hann_MAPKKK10* (rare ZIK) is salt/alkaline-specific, *Hann_MAPKKK132* (conserved *RAF*) is alkaline-specific, and *Hann_MAPKKK26* (conserved *MEKK*) is salt-specific. These findings confirm sub-functionalization of *MAPKKK* genes, as well as multi-stress responsiveness of conserved genes in *H. annuus*.

## 4 Discussion

The mitogen-activated protein kinase (MAPK) cascade constitutes a fundamental signal transduction module in eukaryotes, governing diverse physiological processes ranging from growth and development to environmental stress adaptation (Moustafa et al., 2014). In this study, we identified a total of 1,009 MAPKKK genes across ten Asteraceae genomes. Although family size varies significantly, ranging from 60 in *C. solstitialis* to 139 in *H. annuus*, the lineage generally exhibits a marked expansion compared to other sequenced plant species (Li et al., 2024; Liu et al., 2022; Song et al., 2015; Wang et al., 2023). The expansion observed in the Asteraceae lineage suggests that the proliferation of this gene family may be a key evolutionary strategy contributing to their dominance in diverse ecological niches.

Phylogenetic analysis classified the Asteraceae MAPKKK family into three distinct subfamilies—RAF, MEKK, and ZIK—consistent with classifications reported in bread wheat (Li et al., 2024), grape (Yongqing Feng et al., 2025), and highland bamboo (Xu et al., 2025). This conservation indicates that the divergence of these subfamilies predates the radiation of angiosperms and that their distinct kinase domain architectures have been evolutionarily preserved to fulfill specialized functions. Notably, the RAF subfamily is the most expansive group, accounting for over 50% of the total members and occupying 7 of the 14 phylogenetic clusters (clusters 8–14). This aligns with findings in cucumber and highlights the central role of RAF-like kinases (Wang et al., 2015), which often function as upstream integrators of peptide hormone signals and environmental cues. For example, in *Arabidopsis*, Raf10 and Raf11 have been shown to regulate seed dormancy and ABA responses (Lee et al., 2015), suggesting that the massive expansion of the RAF subfamily in Asteraceae may provide a robust genetic basis for enhanced signal perception and environmental plasticity (Fàbregas et al., 2020).

Gene structure evolution is a primary driver of functional divergence. Our analysis revealed distinct structural features in Asteraceae *MAPKKK* genes, particularly regarding exon-intron organization. While *Arabidopsis* MAPKKKs typically contain fewer exons (Colcombet and Hirt, 2008), sunflower *MAPKKK* genes exhibit a more complex architecture, especially within the RAF and ZIK subfamilies, where most members contain 7–8 exons and some exceed 30. In contrast, the MEKK subfamily retains a compact, intron-poor structure. This structural dichotomy suggests a divergent evolutionary pressure: complex genes may evolve for fine-tuned regulation, while compact genes might satisfy requirements for rapid signal transduction. Similar structural variations have been reported in potato (Shang et al., 2022), kiwifruit (G. Wang et al., 2018), and *Jatropha curcas* (H. Wang et al., 2018), indicating that exon-intron reshuffling is a common theme in MAPKKK evolution.

An interesting finding in our study is the substantial elongation of introns in Asteraceae species. For instance, the average intron length in *Arctium lappa* (1,008 bp) is nearly an order of magnitude longer than that in *Arabidopsis* (~137 bp). This variation is unlikely to be random; rather, it may have profound functional implications. Longer introns are known to harbor a rich repository of cis-regulatory elements, including enhancers, silencers, and non-coding RNA origination sites, which facilitate precise spatiotemporal transcriptional regulation (Monteuuis et al., 2019). Previous studies have established a positive correlation between intron length and regulatory complexity (Heyn et al., 2015). Furthermore, extended introns often increase the potential for alternative splicing, a crucial mechanism for generating proteomic diversity under stress (Bokros et al., 2019; Heyn et al., 2015). Therefore, the structural expansion observed in Asteraceae MAPKKKs, characterized by expanded introns in RAF/ZIK and streamlined coding sequences in MEKK, likely provides the genomic complexity required for fine-tuned regulation in response to fluctuating environmental conditions.

Our comparative genomic analysis provides novel insights into the birth-and-death evolution of the MAPKKK family. We identified 16 orthologous gene groups conserved across all ten species, representing the ancestral conserved gene pool inherited from the common ancestor. Conversely, the identification of lineage-specific OGs (e.g., 14 sunflower-specific groups) points to recent functional specialization. Intriguingly, *C. solstitialis* exhibits a contracted gene repertoire with significant gene losses in several OGs. This phenomenon may reflect an evolutionary strategy of genome streamlining, where redundant genes are purged to optimize resource allocation, a trait often observed in species adapted to specific ephemeral or extreme niches (Magadum et al., 2013).

Mechanistically, WGD serves as the primary engine for this family’s expansion. In nine of the ten studied species, WGD events contributed to ≥30% of the MAPKKK members (23–73 genes per genome), consistent with patterns observed in polyploid species like *Pyropia yezoensis* (Kong et al., 2020) and banana (Wang et al., 2017). These WGD-derived genes preferentially encompass the conserved category, providing genetic redundancy that allows for dosage balance and sub-functionalization while retaining the ancestral gene structure (Soltis et al., 2015). In parallel, DSD plays a distinct role, contributing 21–36 genes per genome. Mediated largely by transposable element activity, DSD often generates intron-poor copies that are translocated to novel genomic contexts (Tan et al., 2021). This aligns with our structural analysis showing that while the conserved RAF/ZIK members possess long introns, a subset of non- conserved genes remain structurally compact. Although initially potentially lacking regulatory sequences, these dispersed copies can acquire new promoters, driving lineage-specific functional diversification (Wang et al., 2012b). The high frequency of DSD in sunflower suggests that transposon-mediated expansion has actively shaped the rare of its stress signaling network, providing an evolutionary testing ground for novel adaptations.

The evolutionary trajectory of the MAPKKK family is shaped by varying selective pressures. Our *K*a/*K*s analysis indicated that the vast majority of syntenic gene pairs have undergone purifying selection (*K*a/*K*s < 1), demonstrating that the fundamental kinase architecture is essential for survival and strictly conserved. The RAF subfamily, particularly the conserved members, exhibits the strongest purifying selection (lowest *K*a/*K*s), reflecting its indispensability in basic cellular processes. However, evolution is dynamic. While we did not detect widespread positive selection (*K*a/*K*s > 1), we observed significantly higher *K*a/*K*s ratios, which indicates relaxed purifying selection, in variable and rare clusters (e.g., MEKK cluster 2) compared to conserved groups. This relaxation of selective constraints is evolutionarily significant. It implies that these duplicate copies are under less pressure to maintain the ancestral sequence rigidly, thereby allowing the accumulation of non-synonymous mutations (Yang et al., 2023). Such evolutionary drift in non-conserved genes provides a genetic reservoir for sub-functionalization or neofunctionalization, enabling Asteraceae species to explore new adaptive landscapes without compromising the integrity of the core signaling network (Fijarczyk and Babik, 2015). This interplay between conserved stability and lineage-specific flexibility explains the evolutionary success of this lineage.

Interestingly, our analysis revealed that rare OGGs exhibit lower median *K*a/*K*s values compared to variable OGGs, a pattern that initially seems counterintuitive given that shared genes are typically more conserved. This phenomenon can be attributed to the recent expansion history of the Asteraceae (Zhang T et al., 2024). A significant portion of these lineage-specific genes originated from recent WGD or transposon-mediated duplication events, particularly in species like *H. annuus*. These young paralogs retain extremely high sequence identity with low levels of *K*s, which mathematically results in suppressed *K*a/*K*s ratios. In contrast, variable OGGs often represent older orthologs that have had sufficient evolutionary time to accumulate both synonymous and non-synonymous mutations, leading to slightly higher *K*a/*K*s values through long-term relaxed selection.

The functional versatility of the MAPKKK family is ultimately reflected in transcriptional responses to environmental stress. Our transcriptomic and qRT-PCR analyses identified specific *Hann_MAPKKK* genes as central hubs in stress signaling. Notably, conserved genes such as *Hann_MAPKKK16* and *Hann_MAPKKK135* were consistently upregulated under drought, salt, and alkaline stresses, mirroring the broad-spectrum stress responses observed in other crops. Comparative analysis reveals both conservation and functional divergence. Similar to cotton *GhMAP4K13*, which positively regulates drought and salt tolerance (Zeng et al., 2025), *Hann_MAPKKK16* was induced >100-fold, suggesting a conserved positive regulatory role. However, functional differentiation is also apparent. Unlike the opposing roles of sugarcane *ShMAPK05* and *ShMAPK07* (Ali et al., 2021), or the differential expression of *SiMAPK3* and *SiMAPKK5* in millet (Zhang et al., 2023), most *Hann_MAPKKK* genes in our study were upregulated. Nevertheless, specific members like *Hann_MAPKKK123* exhibited drought-exclusive responses, indicating a division of labor. This aligns with findings in cassava (Ye et al., 2017) and beet (Zhang et al., 2024), where specific MAPKKK members play distinct roles in stress adaptation. These cross-species comparisons underscore that while the core MAPK signaling architecture is conserved across angiosperms, the specific regulatory roles of individual family members have diversified. The robust and specific expression patterns of sunflower MAPKKKs highlighted in this study provide strong candidate genes for future functional validation and molecular breeding in Asteraceae crops.

## 5 Conclusion

This study presents a comprehensive genomic and evolutionary analysis of the *MAPKKK* gene family across ten Asteraceae species. Through systematic identification and classification, we delineated 1,009 *MAPKKK* genes into three subfamilies (RAF, MEKK, ZIK) and 14 phylogenetic clusters, revealing the complex evolutionary landscape of this signaling family. Comparative genomic analysis further categorized these genes into conserved, variable, and rare groups, highlighting the conserved functional core alongside lineage-specific diversification. Notably, WGD emerged as the primary mechanism driving family expansion, with conserved genes preferentially retained under strong purifying selection, as evidenced by their significantly lower *K*a/*K*s ratios. Structural and promoter analyses underscored subfamily-specific architectural features and a predominance of stress-responsive cis-elements, pointing to the adaptive potential of MAPKKKs. Expression profiling in sunflower under multiple abiotic stresses confirmed both conserved and specialized roles of key *MAPKKK* genes, supporting their involvement in stress signal transduction. Beyond the evolutionary insights, this study identifies several high-priority candidates for molecular breeding. The hyper-responsive conserved MEKK gene *Hann_MAPKKK16* and the late-adaptation ZIK gene *Hann_MAPKKK135* represent ideal targets for enhancing multi-stress resilience in Asteraceae crops. By leveraging these conserved signaling hubs, future breeding programs can potentially synchronize enhanced drought and salinity tolerance in sunflowers and other economically vital Asteraceae species. Collectively, these findings elucidate the evolutionary dynamics, duplication-mediated expansion, and functional diversification of the MAPKKK family in Asteraceae, providing a genetic foundation for understanding stress adaptation and offering candidate genes for future crop improvement.

## Data Availability

The raw data supporting the conclusions of this article will be made available by the authors, without undue reservation.

## Supplementary material

Supplementary Figure 1 Maximum Likelihood phylogenetic tree of *MAPKKK* genes from ten Asteraceae species and *Arabidopsis thaliana*.

Supplementary Figure 2 Correlation between the sizes of MAPKKK families and total of protein coding genes count.

Supplementary Figure 3 *K*a/*K*s analysis of *MAPKKK* genes from ten Asteraceae species and *Arabidopsis thaliana*.

Supplementary Figure 4 Expression patterns of MAPKKK family at drouth stress in *H. annuus*. The heatmap shows the expression levels of *Hann_MAPKKK* genes in leaf tissue. The subfamily and gene category for each *Hann_MAPKKK* member were clearly labeled.

Supplementary Table 1 Taxon list of species used and their genomes information in our analyses.

Supplementary Table 2 Primer sequences used for qRT-PCR validation of candidate genes.

Supplementary Table 3 Summary of *MAPKKK* genes information identified in this study.

Supplementary Table 4 The Count and ratio of gene duplication types across ten Asteraceae species.

Supplementary Table 5 Inter-specific collinear gene pair among ten Asteraceae species.

Supplementary Table 6 Promoter Cis elements identified in all *MAPKKK* genes.

Supplementary Table 7 The expression from the RNA-seq of all *MAPKKK* genes in *Helianthus annuus.*

## References

[B1] Ali A. Chu N. Ma P. Javed T. Zaheer U. Huang M. T. . (2021). Genome-wide analysis of mitogen-activated protein (MAP) kinase gene family expression in response to biotic and abiotic stresses in sugarcane. Physiol. Plant 171, 86–107. doi: 10.1111/ppl.13208, PMID: 32909626

[B2] Ali A. Zhao X. T. Lin J. S. Zhao T. T. Feng C. L. Li L. . (2025). Genome-wide identification and unveiling the role of MAP kinase cascade genes involved in sugarcane response to abiotic stressors. BMC Plant Biol. 25, 484. doi: 10.1186/s12870-025-06490-1, PMID: 40240958 PMC12001561

[B3] Bokros N. Popescu S. C. Popescu G. V. (2019). Multispecies genome-wide analysis defines the MAP3K gene family in Gossypium hirsutum and reveals conserved family expansions. BMC Bioinformatics 20(Suppl 2), 99. doi: 10.1186/s12859-019-2624-9, PMID: 30871456 PMC6419318

[B4] Capella-Gutiérrez S. Silla-Martínez J. M. Gabaldón T. (2009). trimAl: a tool for automated alignment trimming in large-scale phylogenetic analyses. Bioinformatics 25, 1972–1973. doi: 10.1093/bioinformatics/btp348, PMID: 19505945 PMC2712344

[B5] Chen S. (2025). fastp 1.0: An ultra-fast all-round tool for FASTQ data quality control and preprocessing. Imeta 4, e70078. doi: 10.1002/imt2.70078, PMID: 41112039 PMC12527978

[B6] Chen Y.-H. Wang N.-N. Zhang J.-B. Zheng Y. Li X.-B. (2020). Genome-wide identification of the mitogen-activated protein kinase (MAPK) family in cotton (Gossypium hirsutum) reveals GhMPK6 involved in fiber elongation. Plant Mol. Biol. 103, 391–407. doi: 10.1007/s11103-020-00999-9, PMID: 32193788

[B7] Colcombet J. Hirt H. (2008). Arabidopsis MAPKs: a complex signalling network involved in multiple biological processes. Biochem. J. 413, 217–226. doi: 10.1042/bj20080625, PMID: 18570633

[B8] Eddy S. R. (2011). Accelerated profile HMM searches. PloS Comput. Biol. 7. doi: 10.1371/journal.pcbi.1002195, PMID: 22039361 PMC3197634

[B9] El-Gebali S. Mistry J. Bateman A. Eddy S. R. Luciani A. Potter S. C. . (2019). The Pfam protein families database in 2019. Nucleic Acids Res. 47, D427–D432. doi: 10.1093/nar/gky995, PMID: 30357350 PMC6324024

[B10] Fàbregas N. Yoshida T. Fernie A. R. (2020). Role of Raf-like kinases in SnRK2 activation and osmotic stress response in plants. Nat. Commun. 11, 6184. doi: 10.1038/s41467-020-19977-2, PMID: 33273465 PMC7712759

[B11] Feng Y. Li M. Lu S. Shao M. Liang G. Mao J. (2025). Identification of the grape MAPKKK gene family and functional analysis of the VaMAPKKK15 gene under low temperature stress. Plant Physiol. Biochem. 220, 109533. doi: 10.1016/j.plaphy.2025.109533, PMID: 39899958

[B12] Fijarczyk A. Babik W. (2015). Detecting balancing selection in genomes: limits and prospects. Mol. Ecol. 24, 3529–3545. doi: 10.1111/mec.13226, PMID: 25943689

[B13] Group M. (2002). Mitogen-activated protein kinase cascades in plants: a new nomenclature. Trends Plant Sci. 7, 301–308. doi: 10.1016/s1360-1385(02)02302-6, PMID: 12119167

[B14] He W. Zhu Y. Leng Y. Yang L. Zhang B. Yang J. . (2021). Transcriptomic analysis reveals candidate genes responding maize gray leaf spot caused by cercospora zeina. Plants 10. doi: 10.3390/plants10112257, PMID: 34834621 PMC8625984

[B15] Heyn P. Kalinka A. T. Tomancak P. Neugebauer K. M. (2015). Introns and gene expression: cellular constraints, transcriptional regulation, and evolutionary consequences. Bioessays 37, 148–154. doi: 10.1002/bies.201400138, PMID: 25400101 PMC4654234

[B16] Huang X. Sun M. Du X. Quan L. Chao J. Deng X. . (2025). Genome-wide identification of the regulatory network of mitogen-activated protein kinase signaling cascades gene families in Hevea Brasiliensis. BMC Plant Biol. 25. doi: 10.1186/s12870-025-06615-6, PMID: 40316909 PMC12049021

[B17] Huang C. H. Zhang C. Liu M. Hu Y. Gao T. Qi J. . (2016). Multiple polyploidization events across asteraceae with two nested events in the early history revealed by nuclear phylogenomics. Mol. Biol. Evol. 33, 2820–2835. doi: 10.1093/molbev/msw157, PMID: 27604225 PMC5062320

[B18] Jiao Y. Wickett N. J. Ayyampalayam S. Chanderbali A. S. Landherr L. Ralph P. E. . (2011). Ancestral polyploidy in seed plants and angiosperms. Nature 473, 97–100. doi: 10.1038/nature09916, PMID: 21478875

[B19] Jones P. Binns D. Chang H. Y. Fraser M. Li W. Z. McAnulla C. . (2014). InterProScan 5: genome-scale protein function classification. Bioinformatics 30, 1236–1240. doi: 10.1093/bioinformatics/btu031, PMID: 24451626 PMC3998142

[B20] Kim D. Langmead B. Salzberg S. L. (2015). HISAT: a fast spliced aligner with low memory requirements. Nat. Methods 12, 357–360. doi: 10.1038/nmeth.3317, PMID: 25751142 PMC4655817

[B21] Kong F. Cao M. Li N. Sun B. Sun M. Mao Y. (2020). Genome-wide identification, phylogeny, and expressional profiles of the mitogen-activated protein kinase kinase kinase (MAPKKK) gene family in pyropia yezoensis. Front. Mar. Sci. 7. doi: 10.3389/fmars.2020.00193, PMID: 41769693

[B22] Lee S. J. Lee M. H. Kim J. I. Kim S. Y. (2015). Arabidopsis putative MAP kinase kinase kinases Raf10 and Raf11 are positive regulators of seed dormancy and ABA response. Plant Cell Physiol. 56, 84–97. doi: 10.1093/pcp/pcu148, PMID: 25324504

[B23] Li Y. Cai H. Liu P. Wang C. Gao H. Wu C. . (2017). Arabidopsis MAPKKK18 positively regulates drought stress resistance via downstream MAPKK3. Biochem. Biophys. Res. Commun. 484, 292–297. doi: 10.1016/j.bbrc.2017.01.104, PMID: 28131829

[B24] Li M. Li B. Yang M. Wang L. Hou G. Lin Y. . (2022). Genome-wide identification and expression of MAPK gene family in cultivated strawberry and their involvement in fruit developing and ripening. Int. J. Mol. Sci. 23. doi: 10.3390/ijms23095201, PMID: 35563593 PMC9104773

[B25] Li Y. Li Y. Zou X. Jiang S. Cao M. Chen F. . (2024). Bioinformatic identification and expression analyses of the MAPK-MAP4K gene family reveal a putative functional MAP4K10-MAP3K7/8-MAP2K1/11-MAPK3/6 cascade in wheat (Triticum aestivum L.). Plants (Basel) 13. doi: 10.3390/plants13070941, PMID: 38611471 PMC11013086

[B26] Li W. Liu Z. Feng H. Yang J. Li C. (2022). Characterization of the gene expression profile response to drought stress in populus ussuriensis using pacBio SMRT and illumina sequencing. Int. J. Mol. Sci. 23. doi: 10.3390/ijms23073840, PMID: 35409200 PMC8998571

[B27] Li N. Yang Z. Li J. Xie W. Qin X. Kang Y. . (2021). Two VQ proteins are substrates of the osMPKK6-osMPK4 cascade in rice defense against bacterial blight. Rice 14. doi: 10.1186/s12284-021-00483-y, PMID: 33913048 PMC8081811

[B28] Liang Q. Lin X. Liu J. Feng Y. Niu X. Wang C. . (2022). Genome-Wide Identification of MAPKK and MAPKKK Gene Family Members and Transcriptional Profiling Analysis during Bud Dormancy in Pear (Pyrus x bretschneideri). Plants 11. doi: 10.3390/plants11131731, PMID: 35807683 PMC9269224

[B29] Liao X. Shi M. Zhang W. Ye Q. Li Y. Feng X. . (2021). Association analysis of GmMAPKs and functional characterization of GmMMK1 to salt stress response in soybean. Physiologia Plantarum 173, 2026–2040. doi: 10.1111/ppl.13549, PMID: 34487378

[B30] Liu Z. Wang L. Xue C. Chu Y. Gao W. Zhao Y. . (2020). Genome-wide identification of MAPKKK genes and their responses to phytoplasma infection in Chinese jujube (Ziziphus jujuba Mill.). BMC Genomics 21, 142. doi: 10.1186/s12864-020-6548-6, PMID: 32041543 PMC7011567

[B31] Liu X. Zhao M. Gu C. Jiang H. Sun J. Li J. (2022). Genome-wide identification of MAPK family genes and their response to abiotic stresses in tea plant (Camellia sinensis). Open Life Sci. 17, 1064–1074. doi: 10.1515/biol-2022-0466, PMID: 36133426 PMC9462544

[B32] Livak K. J. Schmittgen T. D. (2001). Analysis of relative gene expression data using real-time quantitative PCR and the 2(-Delta Delta C(T)) Method. Methods 25, 402–408. doi: 10.1006/meth.2001.1262, PMID: 11846609

[B33] Magadum S. Banerjee U. Murugan P. Gangapur D. Ravikesavan R. (2013). Gene duplication as a major force in evolution. J. Genet. 92, 155–161. doi: 10.1007/s12041-013-0212-8, PMID: 23640422

[B34] Minh B. Q. Schmidt H. A. Chernomor O. Schrempf D. Woodhams M. D. von Haeseler A. . (2020). IQ-TREE 2: new models and efficient methods for phylogenetic inference in the genomic era. Mol. Biol. Evol. 37, 1530–1534. doi: 10.1093/molbev/msaa015, PMID: 32011700 PMC7182206

[B35] Monteuuis G. Wong J. J. L. Bailey C. G. Schmitz U. Rasko J. E. J. (2019). The changing paradigm of intron retention: regulation, ramifications and recipes. Nucleic Acids Res. 47, 11497–11513. doi: 10.1093/nar/gkz1068, PMID: 31724706 PMC7145568

[B36] Moustafa K. AbuQamar S. Jarrar M. Al-Rajab A. J. Tremouillaux-Guiller J. (2014). MAPK cascades and major abiotic stresses. Plant Cell Rep. 33, 1217–1225. doi: 10.1007/s00299-014-1629-0, PMID: 24832772

[B37] Nakagami H. Pitzschke A. Hirt H. (2005). Emerging MAP kinase pathways in plant stress signalling. Trends Plant Sci. 10, 339–346. doi: 10.1016/j.tplants.2005.05.009, PMID: 15953753

[B38] Nakamura T. Yamada K. D. Tomii K. Katoh K. (2018). Parallelization of MAFFT for large-scale multiple sequence alignments. Bioinformatics 34, 2490–2492. doi: 10.1093/bioinformatics/bty121, PMID: 29506019 PMC6041967

[B39] Pertea M. Pertea G. M. Antonescu C. M. Chang T. C. Mendell J. T. Salzberg S. L. (2015). StringTie enables improved reconstruction of a transcriptome from RNA-seq reads. Nat. Biotechnol. 33, 290–295. doi: 10.1038/nbt.3122, PMID: 25690850 PMC4643835

[B40] Plotnikov A. Zehorai E. Procaccia S. Seger R. (2011). The MAPK cascades: signaling components, nuclear roles and mechanisms of nuclear translocation. Biochim. Biophys. Acta 1813, 1619–1633. doi: 10.1016/j.bbamcr.2010.12.012, PMID: 21167873

[B41] Qiao X. Li Q. H. Yin H. Qi K. J. Li L. T. Wang R. Z. . (2019). Gene duplication and evolution in recurring polyploidization-diploidization cycles in plants. Genome Biol. 20. doi: 10.1186/s13059-019-1650-2, PMID: 30791939 PMC6383267

[B42] Rao K. P. Richa T. Kumar K. Raghuram B. Sinha A. K. (2010). In silico analysis reveals 75 members of mitogen-activated protein kinase kinase kinase gene family in rice. DNA Res. 17, 139–153. doi: 10.1093/dnares/dsq011, PMID: 20395279 PMC2885274

[B43] Ren N. Zhang G. Yang X. Chen J. Ni L. Jiang M. (2024). MAPKKK28 functions upstream of the MKK1-MPK1 cascade to regulate abscisic acid responses in rice. Plant Cell Environ. 47, 5140–5157. doi: 10.1111/pce.15095, PMID: 39166350

[B44] Shang Y. Luo X. Zhang H. Chen M. Yin W. Cao Z. . (2022). Genome-wide identification and analysis of the MAPK and MAPKK gene families in potato (Solanum tuberosum L.). Agronomy 13. doi: 10.3390/agronomy13010093, PMID: 41725453

[B45] Shi H. Hou J. Li D. Hu H. Wang Y. Wu Y. . (2024). Comparative transcriptome and coexpression network analysis reveals key pathways and hub candidate genes associated with sunflower (Helianthus annuus L.) drought tolerance. BMC Plant Biol. 24, 224. doi: 10.1186/s12870-024-04932-w, PMID: 38539093 PMC10976745

[B46] Shi Z. Zhao B. Song W. Liu Y. Zhou M. Wang J. . (2022). Genome-wide identification and characterization of the MAPKKK, MKK, and MPK families in Chinese elite maize inbred line Huangzaosi. Plant Genome 15. doi: 10.1002/tpg2.20216, PMID: 35535627 PMC12807117

[B47] Soltis P. S. Marchant D. B. Van de Peer Y. Soltis D. E. (2015). Polyploidy and genome evolution in plants. Curr. Opin. Genet. Dev. 35, 119–125. doi: 10.1016/j.gde.2015.11.003, PMID: 26656231

[B48] Song Y. Li F. Ali M. Li X. Zhang X. Ahmed Z. F. R. (2025). Advances in protein kinase regulation of stress responses in fruits and vegetables. Int. J. Mol. Sci. 26. doi: 10.3390/ijms26020768, PMID: 39859482 PMC11765796

[B49] Song Q. Li D. Dai Y. Liu S. Huang L. Hong Y. . (2015). Characterization, expression patterns and functional analysis of the MAPK and MAPKK genes in watermelon (Citrullus lanatus). BMC Plant Biol. 15, 298. doi: 10.1186/s12870-015-0681-4, PMID: 26700161 PMC5477810

[B50] Subramanian B. Gao S. Lercher M. J. Hu S. Chen W. H. (2019). Evolview v3: a webserver for visualization, annotation, and management of phylogenetic trees. Nucleic Acids Res. 47, W270–w275. doi: 10.1093/nar/gkz357, PMID: 31114888 PMC6602473

[B51] Sun M. Xu Y. Huang J. Jiang Z. Shu H. Wang H. . (2017). Global identification, classification, and expression analysis of MAPKKK genes: functional characterization of mdRaf5 reveals evolution and drought-responsive profile in apple. Sci. Rep. 7, 13511. doi: 10.1038/s41598-017-13627-2, PMID: 29044159 PMC5647345

[B52] Tan S. Ma H. Wang J. Wang M. Wang M. Yin H. . (2021). DNA transposons mediate duplications via transposition-independent and -dependent mechanisms in metazoans. Nat. Commun. 12, 4280. doi: 10.1038/s41467-021-24585-9, PMID: 34257290 PMC8277862

[B53] Tang H. Krishnakumar V. Zeng X. Xu Z. Taranto A. Lomas J. S. . (2024). JCVI: A versatile toolkit for comparative genomics analysis. Imeta 3, e211. doi: 10.1002/imt2.211, PMID: 39135687 PMC11316928

[B54] Tong C. Jia Y. Hu H. Zeng Z. Chapman B. Li C. (2025). Pangenome and pantranscriptome as the new reference for gene-family characterization: A case study of basic helix-loop-helix (bHLH) genes in barley. Plant Commun. 6, 101190. doi: 10.1016/j.xplc.2024.101190, PMID: 39521956 PMC11783906

[B55] Wang M. Chen J. Zhu X. Tai X. Bo T. (2023). In silico analysis of the MAPK gene family in cabbage and its expression during development and stress response. Horticulturae 9. doi: 10.3390/horticulturae9101119, PMID: 41725453

[B56] Wang H. Gong M. Guo J. Xin H. Gao Y. Liu C. . (2018). Genome-wide identification of jatropha curcas MAPK, MAPKK, and MAPKKK gene families and their expression profile under cold stress. Sci. Rep. 8, 16163. doi: 10.1038/s41598-018-34614-1, PMID: 30385801 PMC6212503

[B57] Wang L. Hu W. Tie W. Ding Z. Ding X. Liu Y. . (2017). The MAPKKK and MAPKK gene families in banana: identification, phylogeny and expression during development, ripening and abiotic stress. Sci. Rep. 7, 1159. doi: 10.1038/s41598-017-01357-4, PMID: 28442729 PMC5430750

[B58] Wang J. Pan C. Wang Y. Ye L. Wu J. Chen L. . (2015). Genome-wide identification of MAPK, MAPKK, and MAPKKK gene families and transcriptional profiling analysis during development and stress response in cucumber. BMC Genomics 16, 386. doi: 10.1186/s12864-015-1621-2, PMID: 25976104 PMC4432876

[B59] Wang Y. Tang H. Debarry J. D. Tan X. Li J. Wang X. . (2012a). MCScanX: a toolkit for detection and evolutionary analysis of gene synteny and collinearity. Nucleic Acids Res. 40, e49. doi: 10.1093/nar/gkr1293, PMID: 22217600 PMC3326336

[B60] Wang G. Wang T. Jia Z. H. Xuan J. P. Pan D. L. Guo Z. R. . (2018). Genome-wide bioinformatics analysis of MAPK gene family in kiwifruit (Actinidia chinensis). Int. J. Mol. Sci. 19. doi: 10.3390/ijms19092510, PMID: 30149559 PMC6164783

[B61] Wang Y. Wang X. Paterson A. H. (2012b). Genome and gene duplications and gene expression divergence: a view from plants. Ann. N Y Acad. Sci. 1256, 1–14. doi: 10.1111/j.1749-6632.2011.06384.x, PMID: 22257007

[B62] Xi X. Hu Z. Nie X. Meng M. Xu H. Li J. (2021). Cross inhibition of MPK10 and WRKY10 participating in the growth of endosperm in arabidopsis thaliana. Front. Plant Sci. 12. doi: 10.3389/fpls.2021.640346, PMID: 33897728 PMC8062763

[B63] Xie Y. Ding M. Zhang B. Yang J. Pei T. Ma P. . (2020). Genome-wide characterization and expression profiling of MAPK cascade genes in Salvia miltiorrhiza reveals the function of SmMAPK3 and SmMAPK1 in secondary metabolism. BMC Genomics 21. doi: 10.1186/s12864-020-07023-w, PMID: 32928101 PMC7488990

[B64] Xie C. Yang L. Gai Y. P. (2023). MAPKKKs in plants: multidimensional regulators of plant growth and stress responses. Int. J. Mol. Sci. 24. doi: 10.3390/ijms24044117, PMID: 36835531 PMC9963060

[B65] Xing K. Zhang J. Xie H. Zhang L. Zhang H. Feng L. . (2024). doi: 10.1007/s11033-024-09551-0, PMID: 38698158

[B66] Xu W. Gao S. Song J. Yang Q. Wang T. Zhang Y. . (2020). NDW, encoding a receptor-like protein kinase, regulates plant growth, cold tolerance and susceptibility to Botrytis cinerea in tomato. Plant Sci. 301. doi: 10.1016/j.plantsci.2020.110684, PMID: 33218645

[B67] Xu L. Guo P. Kuang Y. Su K. Hu K. Gan D. (2025). Characteristics of the MAPK gene family in Zizania latifolia and MAPK3 role in response to fungal pathogen infection. J. Genet. 104. doi: 10.1007/s12041-025-01508-x, PMID: 40919755

[B68] Yang Y. Tang H. Huang Y. Zheng Y. Sun Y. Wang Q. (2023). Genome-wide identification, evolution, and expression analysis of the MAPK gene family in rosaceae plants. Horticulturae 9. doi: 10.3390/horticulturae9121328, PMID: 41725453

[B69] Ye J. Yang H. Shi H. Wei Y. Tie W. Ding Z. . (2017). The MAPKKK gene family in cassava: Genome-wide identification and expression analysis against drought stress. Sci. Rep. 7, 14939. doi: 10.1038/s41598-017-13988-8, PMID: 29097722 PMC5668296

[B70] Yuan G. Sun D. An G. Li W. Si W. Liu J. . (2022). Transcriptomic and metabolomic analysis of the effects of exogenous trehalose on salt tolerance in watermelon (Citrullus lanatus). Cells 11. doi: 10.3390/cells11152338, PMID: 35954182 PMC9367363

[B71] Zeng Q. Peng F. Wang J. Wang S. Lu X. Bakhsh A. . (2025). Identification of the MAP4K gene family reveals GhMAP4K13 regulates drought and salt stress tolerance in cotton. Physiol. Plant 177, e70031. doi: 10.1111/ppl.70031, PMID: 39743670

[B72] Zhang Z. (2022). KaKs_Calculator 3.0: calculating selective pressure on coding and non-coding sequences. Genomics Proteomics Bioinf. 20, 536–540. doi: 10.1016/j.gpb.2021.12.002, PMID: 34990803 PMC9801026

[B73] Zhang P. Duo T. Wang F. Zhang X. Yang Z. Hu G. (2021). *De novo* transcriptome in roots of switchgrass (Panicum virgatum L.) reveals gene expression dynamic and act network under alkaline salt stress. BMC Genomics 22. doi: 10.1186/s12864-021-07368-w, PMID: 33509088 PMC7841905

[B74] Zhang L. Ma C. Kang X. Pei Z. Q. Bai X. Wang J. . (2023). Identification and expression analysis of MAPK cascade gene family in foxtail millet (Setaria italica). Plant Signaling Behav. 18, 2246228. doi: 10.1080/15592324.2023.2246228, PMID: 37585594 PMC10435010

[B75] Zhang X.-M. Wu G.-Q. Wei M. (2024). Genome-wide identification of sugar beet (Beta vulgaris L.) MAPKKKs gene family and their expression in response to salt stress. Sugar Tech 26, 1337–1349. doi: 10.1007/s12355-024-01435-8, PMID: 41770356

[B76] Zhang Z. Xiao J. F. Wu J. Y. Zhang H. Y. Liu G. M. Wang X. M. . (2012). ParaAT: A parallel tool for constructing multiple protein-coding DNA alignments. Biochem. Biophys. Res. Commun. 419, 779–781. doi: 10.1016/j.bbrc.2012.02.101, PMID: 22390928

[B77] Zhang M. Zhang S. (2022). Mitogen-activated protein kinase cascades in plant signaling. J. Integr. Plant Biol. 64, 301–341. doi: 10.1111/jipb.13215, PMID: 34984829

[B78] Zheng W. Yang C. Li W. Bu W. Bu F. (2025). Genome-wide identification, evolution and expression of the CPP gene family in six Theaceae species. Front. Plant Sci. 16. doi: 10.3389/fpls.2025.1700390, PMID: 41293469 PMC12643006

