# Supplementary figures and images for: Comparative genomic analysis of the MAPKKK gene family reveals WGD-driven expansion and strong evolutionary constraints in Asteraceae
Source: Front Plant Sci. 2026 Mar 5;17:1791339. doi: 10.3389/fpls.2026.1791339 (PMC12999904; doi:10.3389/fpls.2026.1791339)

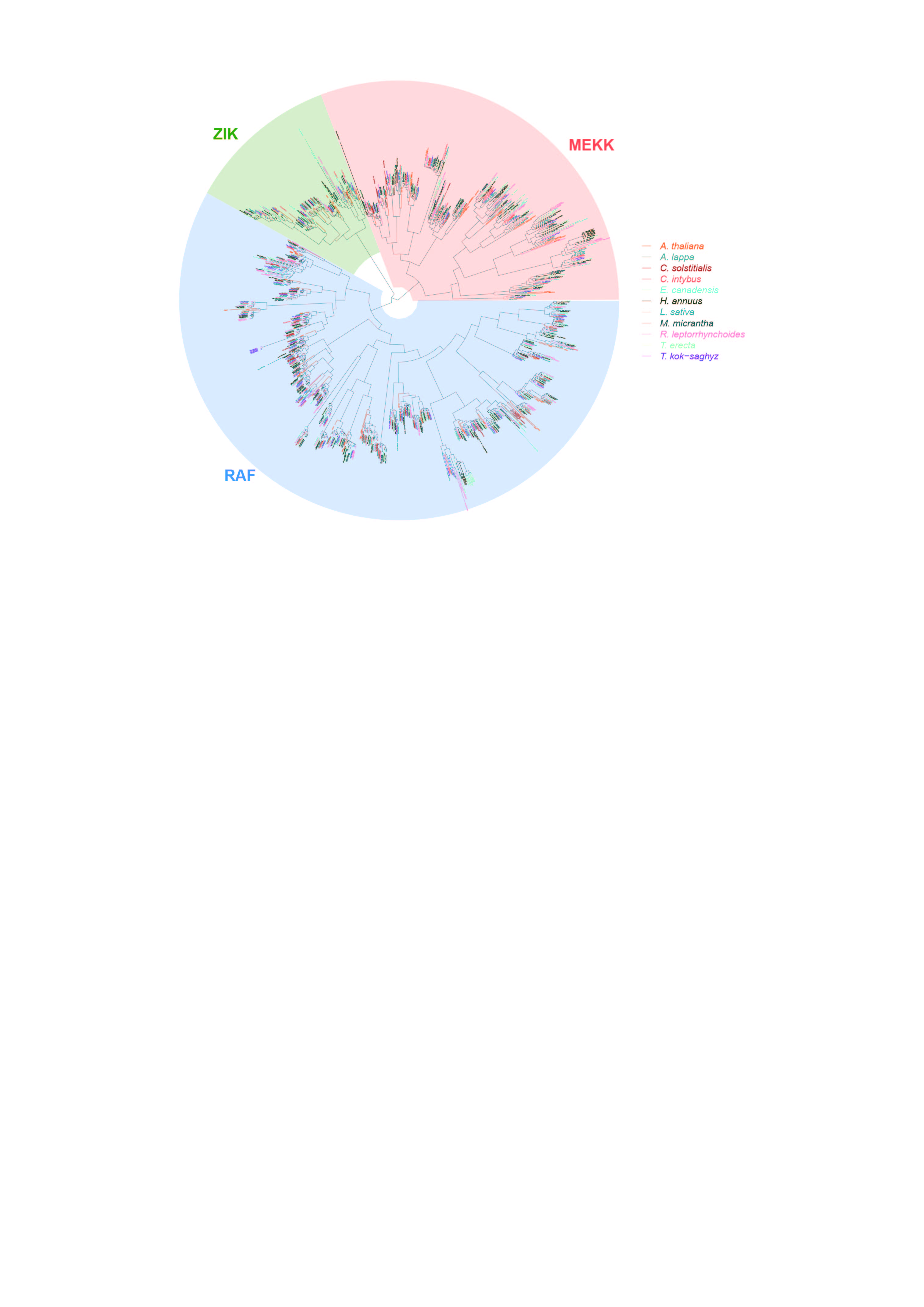

Supplement: Supplementary Figure 1 — Maximum Likelihood phylogenetic tree of MAPKKK genes from ten Asteraceae species and Arabidopsis thaliana. [file Image1.jpeg]

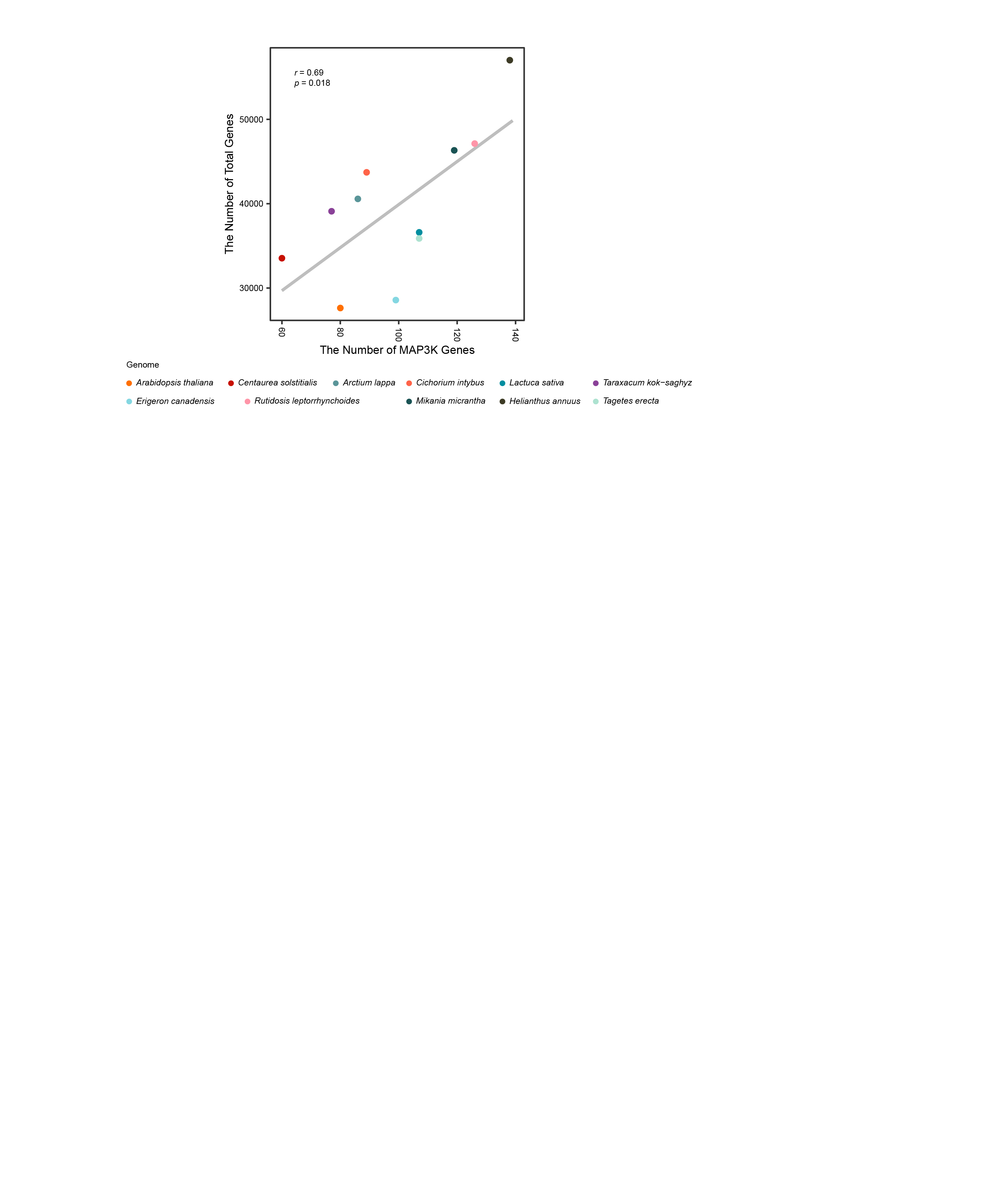

Supplement: Supplementary Figure 2 — Correlation between the sizes of MAPKKK families and total of protein coding genes count. [file Image2.jpeg]

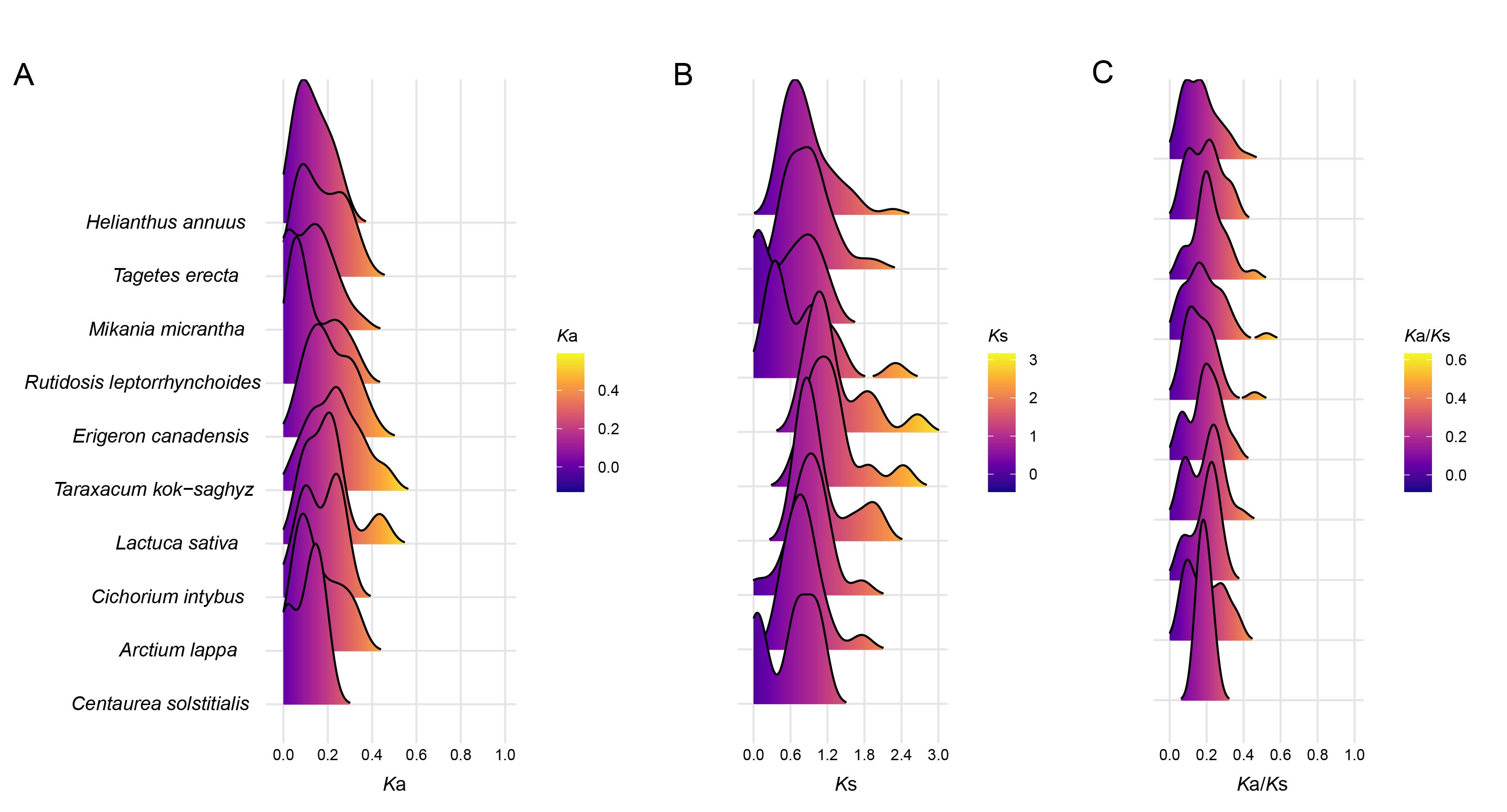

Supplement: Supplementary Figure 3 — Ka/Ks analysis of MAPKKK genes from ten Asteraceae species and Arabidopsis thaliana. [file Image3.jpeg]

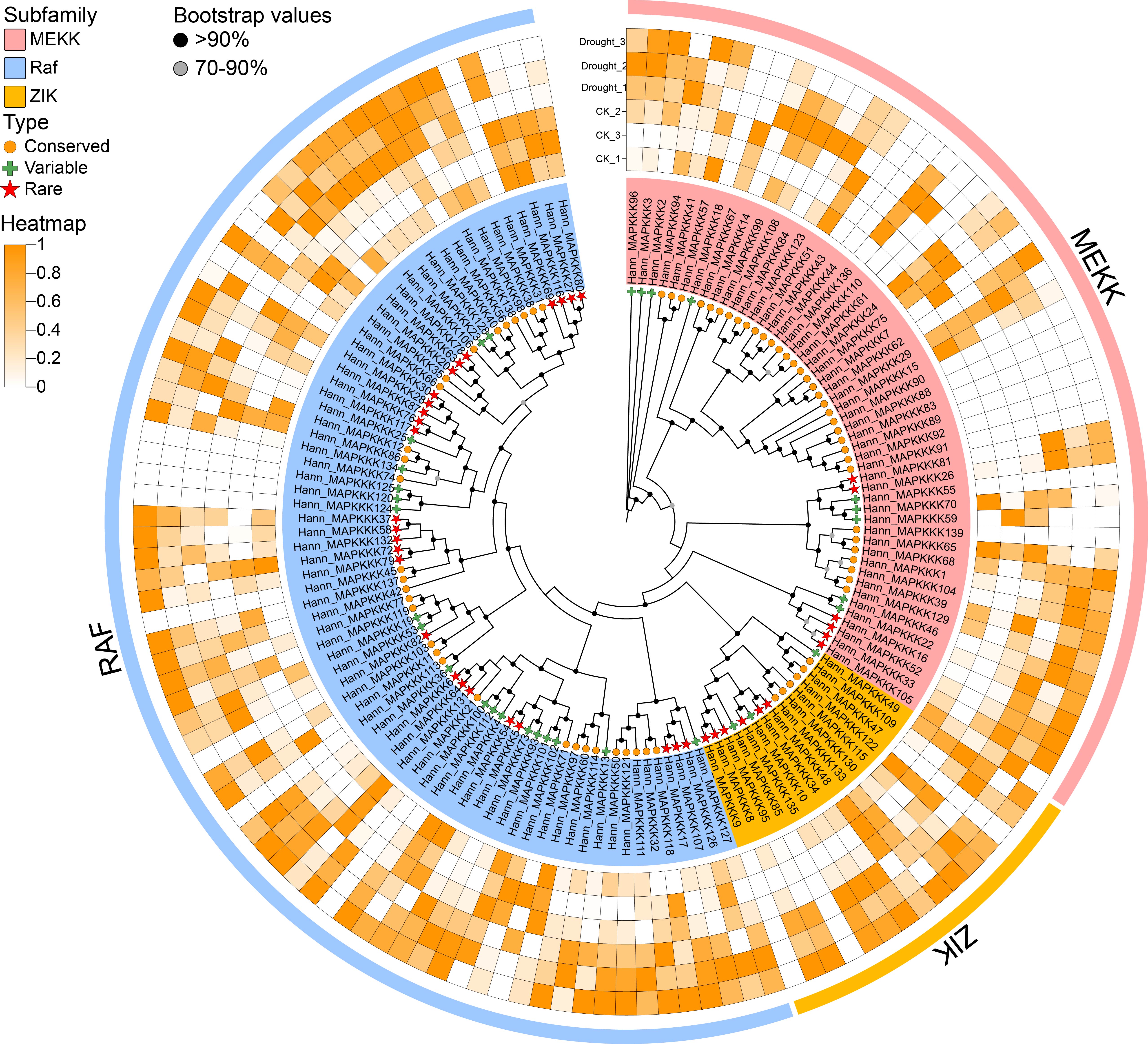

Supplement: Supplementary Figure 4 — Expression patterns of MAPKKK family at drouth stress in H. annuus. The heatmap shows the expression levels of Hann_MAPKKK genes in leaf tissue. The subfamily and gene category for each Hann_MAPKKK member were clearly labeled. [file Image4.jpeg]
